# Constitutive Association of Tie1 and Tie2 with Endothelial Integrins is Functionally Modulated by Angiopoietin-1 and Fibronectin

**DOI:** 10.1371/journal.pone.0163732

**Published:** 2016-10-03

**Authors:** Annamarie C. Dalton, Tomer Shlamkovitch, Niv Papo, William A. Barton

**Affiliations:** 1 Virginia Commonwealth University, Department of Biochemistry and Molecular Biology, Richmond, Virginia, 23298, United States of America; 2 Ben-Gurion University of the Negev, Department of Biotechnology Engineering and the National Institute of Biotechnology in the Negev, Beer-Sheva, 8410501, Israel; Thomas Jefferson University, UNITED STATES

## Abstract

Functional cross-talk between Tie2 and Integrin signaling pathways is essential to coordinate endothelial cell adhesion and migration in response to the extracellular matrix, yet the mechanisms behind this phenomenon are unclear. Here, we examine the possibility that receptor cross-talk is driven through uncharacterized Tie-integrin interactions on the endothelial surface. Using a live cell FRET-based proximity assay, we monitor Tie-integrin receptor recognition and demonstrate that both Tie1 and Tie2 readily associate with integrins α_5_ß_1_ and α_V_ß_3_ through their respective ectodomains. Although not required, Tie2-integrin association is significantly enhanced in the presence of the extracellular component and integrin ligand fibronectin. *In vitro* binding assays with purified components reveal that Tie-integrin recognition is direct, and further demonstrate that the receptor binding domain of the Tie2 ligand Ang-1, but not the receptor binding domain of Ang-2, can independently associate with α_5_ß_1_ or α_V_ß_3_. Finally, we reveal that cooperative Tie/integrin interactions selectively stimulate ERK/MAPK signaling in the presence of both Ang-1 and fibronectin, suggesting a molecular mechanism to sensitize Tie2 to extracellular matrix. We provide a mechanistic model highlighting the role of receptor localization and association in regulating distinct signaling cascades and in turn, the angiogenic switch.

## Introduction

The human vasculature plays a central role in numerous pathological conditions ranging from cardiovascular disease, to macular degeneration, stroke, tumor growth and metastasis [1–4]. Perhaps not surprisingly, many signaling proteins are required for proper vascular development and function. Yet, studies suggest that Vascular Endothelial Growth Factor (VEGF) and the VEGF receptors, the integrins (most notably the fibronectin receptor α_5_ß_1_ and the fibronectin/vitronectin receptor α_V_ß_3_), and the Angiopoietins and Tie receptors are key participants [5,6]. As our understanding of cellular signaling advances, it becomes clear that signaling cascades are significantly more complex than previously appreciated. It is generally accepted that multiple signaling networks are coordinated and co-regulated to control normal physiological processes. Indeed, receptor-receptor interactions on the cell surface can drive changes in receptor conformation, ligand access, and cellular localization, which collectively alter receptor signaling characteristics. This is particularly evident during angiogenesis as Tie, VEGFR, and integrin receptors cross-talk to synchronously govern endothelial cell survival, migration, and proliferation in response to a diverse set of environmental cues [7,8].

It has only recently been appreciated that Tie2 activity is spatially and temporally fine-tuned through its interaction with the functionally related co-receptors Tie1 and integrin cell adhesion receptors α_5_ß_1_ and α_V_ß_3_ [9–12]. The integrin receptors play critical roles in angiogenesis through inside-out and outside-in signaling in response to their extracellular matrix (ECM) ligands. These heterodimeric cell adhesion molecules consist of one α and one ß subunit, the combination of which confers ligand specificity [13]. Activated integrins assimilate signals from the surrounding ECM to modify the rigid actin cytoskeleton inside the cell but may also accept signals from inside the cell to affect their affinity for extracellular ligands [14,15]. At least nine integrin heterodimers exist in endothelial cells, although genetic experiments in mice specifically reveal the essential and compensatory roles of the vitronectin/fibronectin receptor α_V_ß_3_ and fibronectin receptor α_5_ß_1_ in the regulation of angiogenesis [13,16–18].

Integrins are generally believed to influence and modulate the signaling potential of receptor tyrosine kinases including; VEGFR2, PDGFRß, HGF, and Tie2 to list a few [19–21]. For example, α_V_ß_3_ and VEGFR2 associate *in vivo* and sensitize VEGFR2 to VEGF165 in the presence of vitronectin. VEGFR2-dependent activation of the tyrosine kinase c-src directs phosphorylation of the ß_3_ cytoplasmic tail within α_V_ß_3_ promoting interaction between the two cell surface receptors in an inside-out signaling manner. Physical association of α_V_ß_3_ and VEGFR2 is not only critical for receptor sensitization, but also essential for full activation of VEGFR2 [22–25].

In contrast to VEGFR2, the role of integrins in Tie2 signaling is significantly less clear. Tie2 is an endothelial specific receptor tyrosine kinase that signals in response to the angiopoietin ligands. The agonist Angiopoietin-1 (Ang-1) promotes endothelial cell quiescence by clustering Tie2 and initiating pro-survival downstream signaling cascades including the Akt/Survivin pathway [26]. Alternatively, Angiopoietin-2 (Ang-2) is a unique, context-dependent ligand whose function depends on the relative availability of the co-receptor and Tie2 homologue, Tie1 [11]. At high concentrations, Ang-2 behaves as a partial agonist and is capable of activating the Tie2 receptor [27–29]. However, under physiological conditions and in the presence of Tie1, Ang-2 behaves as a receptor antagonist preventing Tie2 activation [11, 27–30]. Additionally, Tie1 appears to counter regulate expression of Tie2 on the cell surface, particularly at tip cells. Therefore, Tie1 not only inhibits Tie2 by directly binding and interfering with active Tie2 clusters, but also inhibits Tie2 signaling via regulation of available protein on the cell surface [11, 31]. Finally, Ang-2 destabilizes the quiescent endothelium, which results in inhibition of Tie2 and vessel regression in the absence of VEGF. Alternatively, in the presence of VEGF, Ang-2 promotes endothelial cell migration and vessel branching [27–28, 32].

Interestingly, both α_V_ß_3_ and α_5_ß_1_ were implicated as additional binding partners of Tie2 and its ligands, Ang-1 and Ang-2 [9–10,12]. For example, Cascone et al. identified a constitutive interaction between α_5_ß_1_ (but not α_V_ß_3_) and Tie2, which was significantly enriched by fibronectin. Intriguingly, integrin association sensitized Tie2 signaling to lower concentrations of Ang-1, demonstrating a uniquely differential response of Tie2 to changes in co-receptor association. However, more recent reports have challenged these initial findings. Thomas et al. describe a transient interaction between Tie2 and α_V_ß_3_ following stimulation with either Ang-1 or Ang-2. The authors further reveal that Ang-2 specifically induces FAK recruitment to the Tie2/integrin receptor complex resulting in integrin internalization and degradation. Under these conditions, Ang-2 is also capable of activating α_V_ß_3_ in endothelial cells independent of Tie2. Indeed, direct binding of the angiopoietin ligands to integrin molecules has precedent in endothelial cells and several non-endothelial cell types lacking Tie2, including; neurons, cardiomyocytes and breast cancer cells. In most cases it remains unclear which ligand, Ang-1 or Ang-2, is the integrin binding partner [33–40].

The potential for receptor complementation in the integrin family makes it difficult to evaluate the role of specific complex components and their influences on Tie receptor signaling, which likely explains many of the conflicting results [18]. Therefore, we used a number of biochemical and biophysical techniques to evaluate the physical basis for individual Tie-integrin and Ang-integrin interactions in an effort to clarify the role of integrins in Tie2 signaling. Surprisingly, using purified signaling components; we demonstrate that both receptor homologues Tie1 and Tie2 associate directly with the endothelial integrins, α_V_ß_3_ and α_5_ß_1_. We further reveal that the amino-terminal ligand-binding domain of the Tie receptors is sufficient and essential for integrin recognition, demonstrating that additional cofactors or ligands are not required for their association. Nevertheless, addition of the α_5_ß_1_ ligand fibronectin significantly stabilizes the integrin/Tie2 complex, raising the intriguing possibility that the extended conformation of the integrin molecules may be critical for Tie2 interactions.

Interestingly, we also reveal that the Ang-1 fibrinogen-like domain (Tie2 receptor binding domain (RBD)), but not that of Ang-2, readily associates with the endothelial integrins both in the presence and absence of Tie2. Finally, we illustrate that integration of both Ang-1 and fibronectin ligands stimulate the integrin-Tie2 complex for selective activation of the mitogen-activated protein (MAP) kinase cascade. Based on our studies, we propose a model for modulation of Tie2 and integrin signaling by ligand association and receptor localization.

## Results

### Tie1 and Tie2 constitutively associate with the endothelial Integrins α_V_ß_3_ and α_5_ß_1_ on the endothelial cell surface

Integrins associate with multiple growth factor receptors at the cell surface to selectively modulate intracellular signaling cascades. Of the nine integrin receptors present in endothelial cells, α_V_ß_3_ and α_5_ß_1_are essential for regulation of the angiogenic response [16, 37]. Knock out mice of the integrins α_V_ and α_5_ phenocopy angiogenic defects of the Tie2 and Ang-1 knockout mice suggesting potential interactions and signaling crosstalk *in vivo* [18]. Nevertheless, integrin receptor compensation has made it difficult to elucidate specific interactions taking place at the endothelial cell surface.

**Fig 1:**
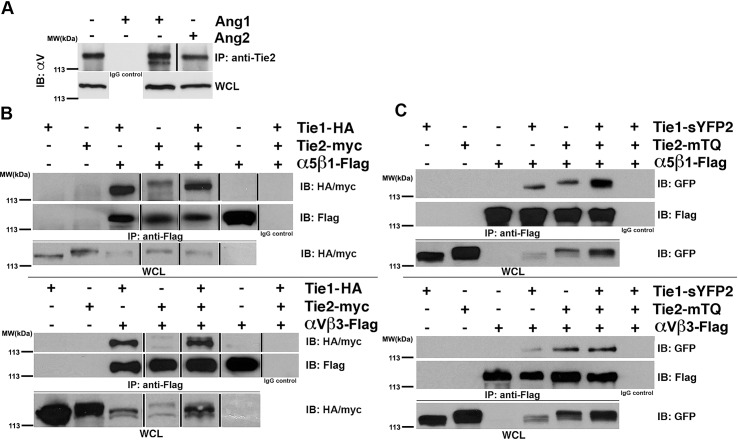
Tie1 and Tie2 interact with the endothelial Integrins α_V_ß_3_ and α_5_ß_1_ through their extracellular domains. (A) Co-immunoprecipitation of endogenous α_V_ with Tie receptor in Ea.hy926 endothelial cells. Cells were serum starved and then treated with vehicle control (PBS), 500 ng/mL rhAng-1 or 500 ng/mL rhAng-2 for 30 minutes at 37 degrees Celsius prior to harvest. α_V_ co-precipitated under all stimulation conditions tested when lysates were incubated overnight with anti-Tie2 antibody, but not when incubated with a non-specific IgG. (B-C) Co-immunoprecipitation of α_V_ß_3_ and α_5_ß_1_ with Tie receptors from transiently transfected HEK293 cells. (B) Full-length integrins α_5_ß_1_ (top panel) or α_V_ß_3_ (bottom panel) were immunoprecipitated with anti-flag antibody, while co-precipitating HA-tagged Tie1 receptor or myc-tagged Tie2 receptor were detected by western blot using anti-HA or anti-myc antibodies, respectively. (C) Same as in (B) alterations in the transfected vectors. Here, Tie1 and Tie2 receptor fluorophore fusion constructs were used in which the intracellular tyrosine kinase domains were replaced with the GFP analogues sYFP2 and mTQ GFP variants respectively. Co-precipitating Tie1 and Tie2 fusion proteins were detected by western blot with anti-GFP antibodies indicating receptor association within the extracellular domains of the proteins (α_5_ß_1_- top panel and α_V_ß3—bottom panel). WCL- Whole Cell Lysate. IP- Immunoprecipitation. IB: Immunoblotting.

Therefore, to more precisely define the combined roles of Tie receptors and integrins, we began by examining the potential of the Tie receptor to associate with endogenous α_V_ß_3_ in endothelial cells using co-immunoprecipitation experiments. Tie protein was immunoprecipitated (using a pan-specific anti-Tie antibody) from protein lysates prepared from confluent EA.hy926 endothelial cells incubated for 30 minutes in the presence of a control vehicle (PBS) or the Tie2 ligands Ang-1 or Ang-2. Western blots were probed for the presence of α_V_, and as illustrated in Fig 1A, antibodies specific to Tie receptors, efficiently precipitate endogenous α_V_ under all conditions examined, while α_V_ is absent from control immunoprecipitates (using a non-specific antibody of the same subclass). Quite surprisingly, we find that the total amounts of precipitated α_V_ fails to significantly change in the presence of either Ang-1 or Ang-2, suggesting that the angiopoietin ligands are not required for integrins and Tie receptor association. It should, however, be noted that we observe an additional faster-migrating α_V_-reactive band that is only apparent in immunoprecipitates from endothelial cells incubated in the presence of Ang-1. This implies that Ang-1, although not having an effect on Tie/integrin association, does have an affect on the modification of Tie2-bound α_V_, which could possibly include heterogeneous glycosylation or proteolytic susceptibility (though we have not investigated further).

Unfortunately, due to the nature of the Tie antibody used for these immunoprecipitation experiments, in was unclear if α_V_ associated with Tie1, Tie2, or both (EA.hy926 as well as many other endothelial cells express both of the highly homologous receptors Tie1 and Tie2) [11]. Thus, to clearly differentiate between specific signaling components of the complex identified above, we utilized transient transfection experiments in HEK293 cells, which lack endogenous Tie1 or Tie2, yet do express α_5_ß_1,_ α_V_ß_3_ and α_V_ß_5_ at low levels [38]. Full-length Tie1, Tie2, and integrin subunits were cloned with either HA, myc, or FLAG epitopes, respectively, and transiently transfected into HEK293 cells. Neither Tie2, nor integrin ligands were added prior to harvest, although it should be noted that there are undoubtedly low levels of integrin ligands including fibronectin present in the media or secreted from the cells themselves. Briefly, integrin heterodimers were immunoprecipitated using anti-FLAG antibody from clarified lysates prepared from confluent cells and probed for the presence of Tie1 or Tie2 using anti-HA or anti-myc antibodies, respectively. Interestingly, and as illustrated in Fig 1B, both Tie1 and Tie2 efficiently co-precipitate when expressed with either α_5_ß_1_ (top panel) or α_V_ß_3_ (bottom panel) in HEK293 cells. Furthermore, in agreement with our earlier experiments, Tie/integrin association occurs in the absence of Ang-1 or Ang-2 stimulation.

To further understand the physical basis for this phenomenon, we evaluated the hypothesis that receptor recognition involved their respective ectodomains. To examine this possibility, we replaced the intracellular tyrosine kinase domain of the Tie receptors with fluorophore variants of GFP and repeated the co-immunoprecipitation experiments described above. Previous studies have demonstrated that Tie2-GFP receptor chimeras, which retain the full ectodomain and trans-membrane portion of the protein, are functionally similar to full length Tie receptors as demonstrated by Tie2-GFP’s ability to cluster in response to its agonist, Ang-1 [11,30]. As shown in Fig 1C, Tie1-GFP and Tie2-GFP receptor chimeras (lacking their respective cytoplasmic domains) readily co-immunoprecipitate with either full-length flag-tagged α_5_ß_1_ (top panel) or α_V_ß_3_ (bottom panel) integrins. Collectively, our results illustrate that Tie/integrin recognition occurs between their respective ectodomains and further suggest that although Ang ligands do not dramatically affect Tie/integrin interactions, at least in this context, they may potentially alter receptor localization and/or cellular signaling.

### The Tie receptors associate directly with either α_V_ß_3_ and α_5_ß_1_ and the Tie2/integrin complex is stabilized by fibronectin

**Fig 2:**
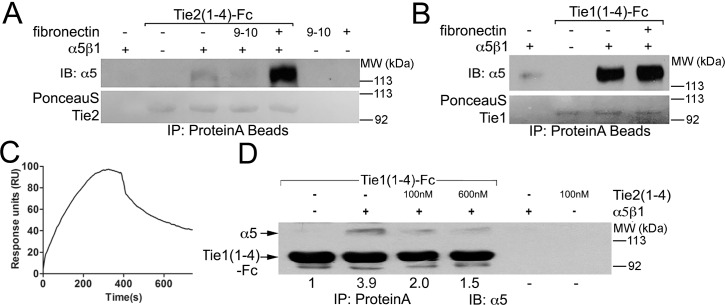
Direct association between Tie1 and Tie2 and integrins using purified proteins. (A) Purified Tie2(1–4)-Fc protein (33nM) was incubated with purified α_5_ß_1_ ectodomain protein (12.5nM) in the presence or absence of either full-length or recombinant fibronectin (5μg/mL) containing only the 9th and 10th fibronectin type-III repeats (9–10). All purified components were present at the indicated concentrations in 1mL of HBST buffer and incubated overnight with 25μL ProteinA resin. Following precipitation of Tie2(1–4)-Fc with Protein-A sepharose, proteins were electrophoresed by SDS-PAGE, transferred to nitrocellulose, and Tie2 was briefly visualized with PonceauS prior to western blotting with α_5_-specific antibodies. The α_5_ß_1_ ectodomain readily precipitates with Tie2, although the addition of full-length fibronectin, but not recombinant, truncated fibronectin (9–10), greatly enhances the Tie2/integrin interaction. (B) As in (A) using Tie1(1–4)-Fc protein (33nM) incubated with purified α_5_ß_1_ ectodomain protein (12.5nM) in the presence or absence of full-length fibronectin (5μg/mL). Unlike the experiments shown in (A), fibronectin does not significantly enhance the Tie1/integrin interaction. (C) Surface Plasmon Resonance depiction of purified and untagged α_5_ß_1_ binding to immobilized Tie1 (Fc tagged full extracellular domain protein). Similar experiments were completed with immobilized Tie2, however no interaction was captured. (D) 33nM purified Tie1(1–4)-Fc protein was incubated with purified α_5_ß_1_ ectodomain (12.5nM) in the presence or absence of an increasing amount of Tie2(1–4) untagged protein (either 100nM or 600nM as indicated). Following precipitation of Tie1(1–4)-Fc with Protein-A sepharose, proteins were subjected to western blotting with α_5_-specific antibodies. α_5_ß_1_ readily precipitates with Tie1, demonstrating that the Ig and EGF repeats are sufficient for α_5_ß_1_ recognition. Furthermore, Tie2 (1–4) effectively competes with Tie1 for integrin binding, suggesting a shared binding interface of Tie1 and Tie2 on the integrin molecule. Quantified values represent a normalized ratio of α_5_ to Tie1(1–4)-Fc.

To more precisely define the requirements for integrin association, and exclude the possibility that an indirect binding partner mediates the Tie/integrin interactions, we utilized purified protein components in an *in vitro* binding assay. Individual receptor ectodomains, or variants of Tie1, Tie2, α_V_ß_3,_ α_5_ß_1_, and Ang ligands were expressed and purified from mammalian cells. Briefly, proteins were expressed as Fc fusion proteins from stably transfected HEK293 cells, purified via affinity chromatography on Protein-A Sepharose and in some cases cleaved from their affinity tag via thrombin or TEV proteolysis, followed by gel filtration chromatography. Representative purified proteins were visualized by coomassie stain in S1 Fig. Purified α_5_ß_1_ (12.5nM) was incubated with either the extracellular ligand-binding domain of Tie2-Fc (33nM) (excluding the fibronectin repeats—designated Tie2 (1–4)) (Fig 2A) or the equivalent region of Tie1 (33nM) (excluding the fibronectin repeats—designated Tie1 (1–4)) (Fig 2B), in the presence or absence of recombinant fibronectin (5μg/mL) prior to precipitation of the Fc-tagged proteins with Protein-A Sepharose.

In agreement with previous experiments, in the presence of either purified Tie1-Fc or Tie2-Fc, α_5_ß_1_ is co-precipitated, illustrating that these receptors proteins interact directly (See Fig 2A and 2B). Furthermore, because the Tie receptor variants are lacking their membrane proximal fibronectin type-III repeats, our data also reveals that the integrins recognize the compact Tie2-ligand binding domain (or equivalent region of Tie1) containing the three Ig and three EGF repeats. Interestingly, when the experiments are repeated in the presence of full-length fibronectin, the amount of co-precipitating α_5_ß_1_ increases dramatically for Tie2, but remains relatively constant for Tie1 (See Fig 2A and 2B, and S2 Fig). Alternatively, when a recombinant protein containing only the 9th and 10th Fibronectin Type III repeats is added (FN9-10), which retains the principal fibronectin RGD motif that efficiently interacts with α_5_ß_1_, the Tie2/integrin association is unaffected (See Fig 2A). These results demonstrate that unlike Tie1, Tie2 is sensitive to the presence of full-length fibronectin. Additionally, using Surface Plasmon Resonance, we were able to detect binding of purified α_5_ß_1_ to immobilized Tie1 protein as shown in Fig 2C. Similar experiments were completed with immobilized Tie2 protein; however, no binding was detected. Collectively, we interpret this to indicate that fibronectin facilitates associate between α_5_ß_1_ and Tie2, yet does not have an effect on Tie1/integrin binding. Furthermore, fibronectin likely binds α_5_ß_1_ and Tie2 simultaneously, utilizing a binding site for Tie2 outside of the 9th and 10th FNIII domains of fibronectin.

To further evaluate whether the Tie receptors interact with α_5_ß_1_ using the same, or overlapping molecular interface, we examined the effect of excess untagged Tie2 on formation of Tie1-Fc/ α_5_ß_1_ complexes. Specifically, varying concentrations of Tie2 (either 100nM or 600nM) were co-incubated with Tie1-Fc (33nM) and α_5_ß_1_ (12.5nM) and analyzed as described above. Interestingly, as demonstrated in Fig 2D, we reveal that increasing the Tie2 concentration results in a dramatic decrease in the amount of α_5_ß_1_ precipitated with Tie1-Fc, implying that Tie2 effectively competes with Tie1 for α_5_ß_1_ binding and that they likely utilize the same (or overlapping) receptor interface for integrin recognition. Collectively, these results establish that additional cellular components are not required for Tie/integrin complex formation while also precisely defining the requirements for the Tie Ig and EGF domains in integrin recognition.

### The Ang-1 receptor binding domain, but not that from Ang-2, directly associates with both α_V_ß_3_ and α_5_ß_1_ independently of Tie2

**Fig 3:**
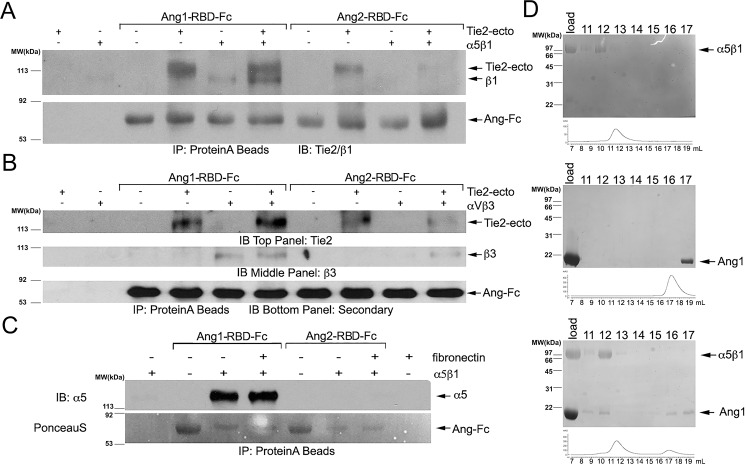
Direct association between the Ang-1 receptor binding domain and α_V_ß_3_ and α_5_ß_1_. (A) The Ang-1 and Ang-2 receptor binding domains were expressed and purified as Fc fusion proteins, and incubated at 50nM with purified α_5_ß_1_ (12.5nM) in the presence or absence of the Tie2 ectodomain (33nM). Precipitating proteins were visualized with Tie2 and ß_1_ -specific antibodies (α_5_ migrates at the same position as Tie2 and, therefore, α_5_-antibodies could not be used). The Tie2 ectodomain readily precipitates with both the Ang-1 or Ang-2 receptor binding domains, while α_5_ß_1_ only precipitates with Ang-1-RBD. (B) Similar to the experiment in (A) using purified ectodomain of α_V_ß_3_ protein incubated with either Ang-1-RBD-Fc or Ang-2-RBD-Fc. Precipitating α_V_ß_3_ was visualized with antibodies specific to ß_3_. (C) The Ang-1 and Ang-2 receptor binding domain Fc fusion proteins (50nM) were incubated with purified α_5_ß_1_ (12.5nM) in the presence or absence of human fibronectin. Following precipitation of Ang-Fc proteins with Protein-A sepharose, proteins were electrophoresed by SDS-PAGE, transferred to PVDF, and briefly visualized with PonceauS prior to western blotting with α_5_-specific antibodies. The α_5_ß_1_ ectodomain readily co-precipitates with Ang-1-RBD, but not with Ang-2-RBD. Fibronectin does not appear to significantly influence the Ang-1/integrin interaction. (D) Direct association between Ang-1-RBD (10μM) and α_5_ß_1_ (5μM) by size-exclusion chromatography. Indicated fractions were incubated in loading buffer with BME for 5 minutes and resolved on a 10% SDS-PAGE gel to separate proteins eluting from the column in reducible crosslinked complexes. Top panel: Purified α_5_ß_1_ ectodomain was chromatographed on a Superdex 200 column and elutes as a single peak at ~12 mL. Middle panel: Purified Ang-1-RBD elutes from a Superdex 200 as a single peak at ~17 mL. Bottom panel: 5μM α_5_ß_1_ purified protein was cross-linked to a 2 molar excess of Ang-1-RBD using DTSSP and separated on a Superdex 200 column. Under these conditions, Ang-1-RBD now elutes in a complex with α_5_ß_1_ at ~11.5 mL.

Previous studies have suggested an integrin-dependent yet Tie2-independent function for angiopoietin ligands in endothelial cells [33,37,39]. Interestingly, the effects of angiopoietins also have been described in cell types that lack Tie2, the primary endothelial receptor for the angiopoietin ligands, including cardiomyocytes, breast cancer cells, and neurons [34–36,38,40]. Therefore, to further investigate the association of integrins with the Ang ligands, we repeated our *in vitro* binding assay using purified Fc-tagged receptor-binding domains (RBD) of Ang-1 and Ang-2 (the receptor binding domain is equivalent to the fibrinogen-like domain of the angiopoietins and does not include the coiled coil or super clustering domains of the ligands). Purified angiopoietin-RBD proteins (50nM) were incubated with either the ectodomains of α_V_ß_3_ or α_5_ß_1_ (12.5nM) in the absence and presence of the Tie2 ectodomain (24nM). As a positive control, we incubated Ang-1-RBD and Ang-2-RBD ligand with Tie2 to demonstrate their functional ability to associate with the receptor. As demonstrated in Fig 3A and 3B, the Ang-1-RBD-Fc fusion protein, but not Ang-2-RBD-Fc, readily precipitates integrin protein. Interestingly, Ang1-RBD-Fc is capable of binding to the integrin proteins regardless of the presence of Tie2, indicating that integrins independently associate with Ang-1-RBD and the Tie receptors. Moreover, the presence of Tie2 does not interfere with the ability of Ang-1-RBD to bind to either α_V_ß_3_ or α_5_ß_1._

To further evaluate the role of fibronectin on this interaction, we repeated our binding assays with Ang-1-RBD-Fc and Ang-2-RBD-Fc fusion proteins (50nM) incubated with α_5_ß_1_ integrin (12.5nM) in the presence or absence of fibronectin (5μg/mL). As demonstrated in Fig 3C, we reveal that the amount of α_5_ß_1_ that co-precipitates with Ang-1-RBD fails to significantly change in the presence or absence of fibronectin, while Ang-2-RBD remains unable to co-precipitate with purified α_5_ß_1_ independent of fibronectin. Finally, to further demonstrate Ang-1-RBD’s ability to associate with α_5_ß_1_ in isolation, a molar excess of Ang1-RBD was crosslinked to purified α_5_ß_1_ using DTSSP and separated using gel filtration chromatography. Chromatographic fractions were subsequently analyzed by SDS-PAGE. As observed in Fig 3D, Ang1-RBD binding to α_5_ß_1_ induces a significant shift in the elution profile of Ang1-RBD (compared to the elution profile of Ang1-RBD alone), consistent with the formation of a 1:1 complex. Complex formation may also be observed in the absence of cross-linker, although the decreased yield suggests that it remains less stable under these conditions (DTSSP is utilized to stabilize the complex for structural studies).

### The Tie2 / α_5_ß_1_ complex is stabilized by fibronectin in live cells

As discussed earlier, addition of fibronectin enhances the amount of Tie2-associated integrin precipitated in our co-IP assays suggesting that it stabilizes Tie/integrin interactions. Therefore, to further validate our findings and probe the potential role of fibronectin on Tie-integrin association, we followed the interaction of Tie2 and α_5_ß_1_ in the presence or absence of fibronectin by Förster Resonance Energy Transfer (FRET) microscopy in live cells. FRET microscopy is a particularly powerful approach for examining real time protein-protein interactions and has been utilized extensively in our lab to monitor interactions with Tie2 [11,30]. HEK293 cells were plated on either vehicle or fibronectin coated culture dishes, transiently transfected with GFP-fused receptor constructs, and imaged between 24 and 48 hours post-transfection. Specifically for these studies, α_5_-YFP, ß_1_-mCherry, and Tie2-mTurquoise (mTQ) were transiently co-transfected into HEK293 cells and protein localization and receptor interactions were followed via fluorescence under various conditions. The fluorophore variants of CFP (mTurquoise/mTQ) and YFP (mYFP or sYFP2) were utilized for their increased intensity, and in the case of mTQ, display a single exponential decay profile, which greatly simplifies fluorescence lifetime (FLIM)-FRET measurements. We noted that co-expression of ß_1_ significantly enhances surface expression of α_5_-mYFP, and therefore, ß_1_-mCherry was routinely co-transfected along with α_5_.

**Fig 4:**
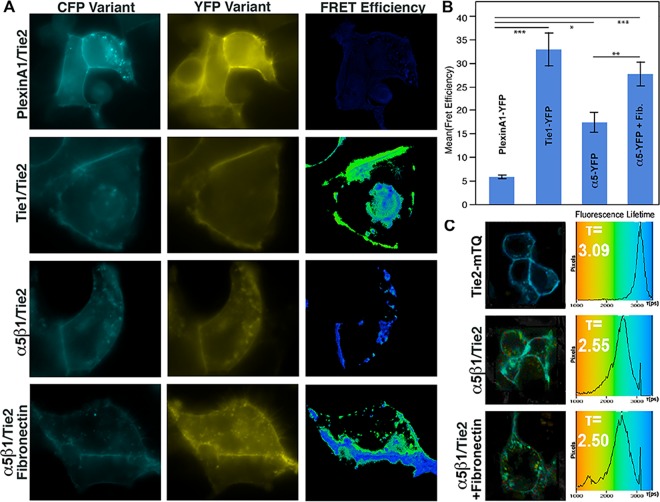
Fibronectin increases Tie-integrin association by sensitized emission FRET and FLIM-FRET. (A) HEK293 cells were transiently transfected with vectors for the indicated receptor fusion proteins and visualized by fluorescence microscopy. FRET images were obtained for sensitized emission calculations on a wide-field microscope and representative images are shown. PlexinA1 does not associate with Tie2 and is, therefore, used as a negative control. Alternatively, Tie1 and Tie2 readily associate on the cell membrane and, therefore, serve as a positive control. FRET values for n>3 experiments (displayed graphically in (B)), and demonstrate a robust interaction between Tie2 and α_5_ß_1_. Values are depicted +/- Standard Error. * indicates student t-test values of p < 0.05, ** p<0.01, *** p<0.001 (C) Fluorescence lifetime (FLIM-FRET) measurements were evaluated to corroborate the values obtained by sensitized emission using a Becker & Hickl TCSPC system. FLIM-FRET controls not shown here are available in S3 Fig.

The positive control Tie1-sYFP2/Tie2-mTQ was used to validate our experimental system. When the two receptors are co-expressed, as illustrated in Fig 4A and 4B, we observe an average FRET efficiency of 32.9% +/- 5.5% using sensitized emission on a wide-field fluorescence microscope, which is comparable to our previously published results [11]. As a negative control, we utilize the unrelated receptor PlexinA1 fused to YFP along with Tie2-mTQ. For these receptors, which fail to associate on the cell surface, we observe a significantly decreased FRET efficiency of 5.8% +/- 0.4%. Alternatively, when Tie2-mTQ and α_5_-YFP are co-expressed and imaged on standard culture dishes, we observe a FRET efficiency of 17.4% +/- 2.1%, significantly above our negative control value, yet approximately half of the signal obtained for Tie1 and Tie2 (See Fig 4B). Interestingly, however, and in agreement with our co-immunoprecipitation experiments, we obtain a significantly higher FRET efficiency of 27.7% +/-2.5% when the cells are cultured on fibronectin. Thus, unlike the angiopoietin ligands, and in agreement with our *in vitro* binding experiments, fibronectin appears to dramatically affect and stabilize Tie2/α_5_ß_1_ interactions.

To corroborate our results obtained using sensitized emission, we utilize Fluorescence Lifetime Microscopy (FLIM-FRET) and measure the exponential decay of mTQ fluorescence using Time Correlated Single Photon Counting (TCSPC) with a femtosecond mode-locked Ti:Sapphire laser. In the presence of FRET, the fluorescence lifetime (τ) of the donor fluorophore decreases substantially and correlates with FRET findings obtained by other methods. Using conditions, which do not appreciably induce fluorophore bleaching, Tie2-mTQ was determined to display a single exponential mean fluorescence lifetime of 3.09 ns (See Fig 4C). Similar values (3.00 ns) were obtained when Tie2-mTQ was co-expressed with the non-interacting negative control Plexin-A1-YFP. In contrast, when Tie2-mTQ is co-expressed with our positive control (Tie1-YFP), we observed a reduced two-component mean lifetime of 2.3 ns, demonstrating significant energy transfer from the donor to acceptor due to close proximity of the receptors (Shown in S3 Fig). Likewise, in the presence of α_5_-YFP and in agreement with our sensitized emission experiments, the Tie2-mTQ mean fluorescence lifetime decreased to 2.55 ns when HEK293 cells were cultured on untreated dishes. Addition of 5 μg/mL fibronectin protein coated on the imaging dishes, reduced the lifetime slightly more to 2.5 ns corroborating our earlier FRET results obtained using sensitized emission (See bottom panel of Fig 4C).

A similar interaction was observed between Tie1-mTQ and α_5_-YFP/ß_1_-mCherry using acceptor photobleaching FRET microscopy. Similar to sensitized emission, acceptor photobleaching is an alternate method to measure FRET that is just as reliable and sensitive as the others and has been routinely used to monitor Tie2 interactions in the past. Under these conditions, our positive control (Tie1-sYFP2/Tie2-mTQ) yielded a FRET efficiency of 16.8% +/- 1.1%, which is similar to that obtained by other methods (S3C and S3D Fig). Interestingly, and in agreement with our earlier results, we find that when co-expressed, Tie1 and α_5_ show a significant FRET efficiency (11.0%+/- 1.3%) compared to the PlexinA1 negative control (3.4% +/- 1.8%), demonstrating that Tie1 and α_5_ß_1_ interact directly on the cell surface. Thus, FRET microscopy confirms that the interaction between Tie2 and integrin is enhanced by the addition of exogenous fibronectin.

### Receptor cross-talk modulates MAPK signaling

The integrin and Tie receptors activate distinct signaling cascades including Focal Adhesion Kinase (FAK), MAPK (ERK1/2), and Akt, which individually elicit diverse physiological effects on endothelial cells. In an effort to identify the downstream effects brought about by integrin/Tie interactions, we monitored activation of these signaling pathways following exposure of Telomerase-Immortalized Microvascular Endothelial (TIME) cells to either the Tie2 receptor ligands, Ang-1 and Ang-2, or the ECM component and integrin agonist fibronectin. Briefly, suspended TIME cells were incubated with shaking for one hour in serum and growth factor-free EBM-2 media and plated with or without Angiopoietin ligand on PBS-treated plates or plates that had been previously coated with fibronectin. To follow activation of specific signaling cascades, we monitored FAK, MAPK (ERK1/2), and Akt protein phosphorylation in western blots of cellular lysates harvested following a brief 15 minute incubation (longer time points are shown in S4C Fig).

**Fig 5:**
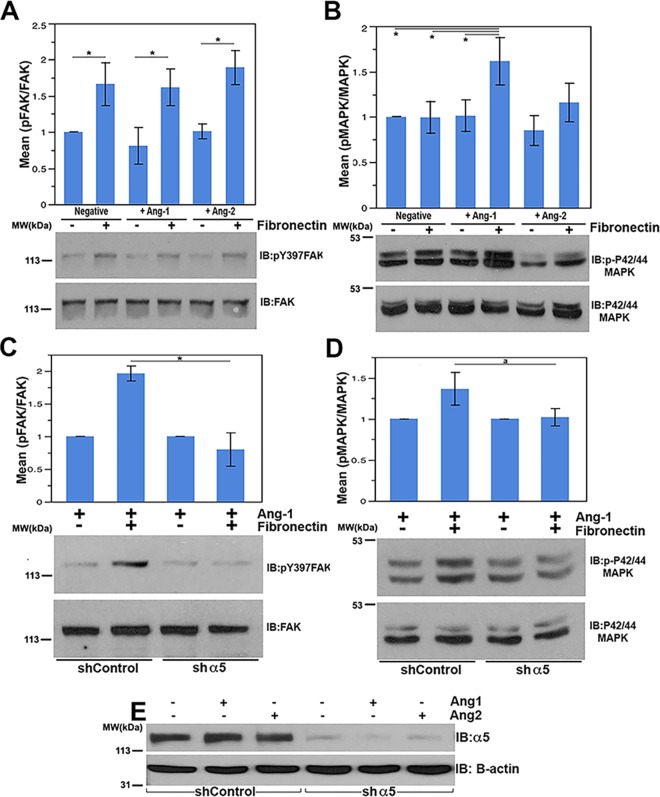
MAPK is cooperatively regulated by the Tie2/Integrin complex in response to Ang-1 and fibronectin. Serum starved telomerase-immortalized endothelial cells (TIMEs) were plated for 15 minutes on control treated or fibronectin treated dishes with vehicle stimulation, 500 ng/mL full length Ang-1, or 500 ng/mL full length Ang-2. Cell lysates were resolved by SDS-PAGE and analyzed for p-FAK (Y397) / FAK (A) and p-ERK1/2 (T202/Y204)/ ERK (B) after treatment. (C and D) Integrin α_5_ protein levels were decreased using shRNA in TIME cells. (C) Ang-1 stimulated shControl cells or sh-α_5_ TIME cells were plated on either PBS treated or 5 μg/mL fibronectin treated plates, lysed, and probed as above for p-FAK (Y397) / FAK (C) or p-ERK1/2 (T202/Y204) / ERK1/2 (D). (E) Levels of α_5_ protein were monitored in control and silenced cells via western blotting. Actin was used as a loading control. Error bars represent the standard error of 3 independent experiments. * represents student t-test values of p < 0.05.

FAK is a well-characterized partner of integrin molecules that primarily regulates actin cytoskeletal rearrangements, facilitating motility and cell spreading. Integrin activation of FAK can be robustly followed using antibodies against tyrosine-phosphorylated FAK residue Y397 [41]. As shown in S4A Fig and Fig 5A, cells plated on fibronectin demonstrated a consistent and significant increase in Y397 phosphorylation of FAK. Alternatively, the presence or absence of angiopoietin ligand had little effect on FAK activation. Similarly, the combination of either Ang-1 or Ang-2 in addition to fibronectin had no effect over that seen with fibronectin alone at 15 minutes. Finally, to verify FAK activation is dependent on α_5_ß_1_, we efficiently reduced the levels of α_5_ protein by lentiviral-mediated shRNA delivery, as demonstrated in Fig 5E. As expected, cellular FAK phosphorylation was not significantly stimulated above basal levels in response to fibronectin in α_5_-silenced cells (See Fig 5C). Thus, integrin activation, at least evaluated by FAK signaling, does not seem significantly modulated by the Tie2 ligands Ang-1 or Ang-2 under these conditions.

We next examined the Akt signaling cascade for activation by integrins and Tie2 [42]. First, we examined Akt activation in response to the Tie2 ligands Ang-1 and Ang-2. As expected, cells cultured in the presence of Ang-1 display elevated levels of phosphorylated Akt (using anti-pT308 antibodies) (S5B Fig). Alternatively, levels of activated Akt were not significantly affected in endothelial cells stimulated with Ang-1 yet expressing diminished levels of α_5_ (S5B Fig). Additionally, levels of activated Akt did not increase in the presence of fibronectin alone; therefore, we did not examine Akt further (See S5A Fig).

Finally, MAPK activation was specifically monitored by western blotting with anti-phospho-MAPK antibodies [43]. As shown in Fig 5B and S5C Fig, neither the Tie2 ligands Ang-1 or Ang-2, nor the α_5_ß_1_ ligand fibronectin alone significantly affect MAPK activation at the 15 minute time point in our assay. At later time points, fibronectin treatment induces a slight increase in MAPK phosphorylation while Ang-1 has no significant effect (See S5B Fig). Interestingly, at the 15 minute time point, only the combination of Ang-1 and fibronectin is capable of significantly stimulating MAPK phosphorylation. Alternatively, neither Ang-2 nor the combination of Ang-2 and fibronectin significantly affect MAPK activation.

Importantly, our results are reminiscent of prior studies demonstrating that α_5_ß_1_ can promote prolonged MAPK signaling downstream of VEGFR2 in the presence of matrix-associated VEGF. Likewise, although the absolute change in MAPK stimulation is modest in the presence of both Ang-1 and fibronectin (~1.6 fold), the relative level is consistent with that observed for matrix-bound VEGF stimulation of MAPK by VEGFR2 and α_5_ß_1_ reported by Chen *et al*. (~1.7 fold) [25].

To further clarify the role of α_5_ß_1_ in this process, we repeated the assay using either control or α_5_-silenced endothelial cells as described above. Once again, we observe an increase in MAPK phosphorylation when control cells (shControl) expressing a scrambled shRNA construct are stimulated with both Ang-1 and fibronectin. Alternatively, when α_5_ protein levels are substantially decreased, we fail to observe an increase in MAPK activation following Ang-1 and fibronectin addition (See Fig 5D). Therefore, under these conditions, α_5_ß_1_ activation is required to stimulate MAPK signaling in response to Ang-1 and fibronectin.

Finally, to identify the functional significance of our activity assays, we followed endothelial cell adhesion in the presence and absence of fibronectin, Ang-1, or the combination of the two ligands. Interestingly, we find that although adhesion is unaffected in the presence of either ligand alone, the combination of fibronectin and Ang-1 significantly increases the number of adherent cells at 30 minutes. Indeed, under experimental conditions comparable to the aforementioned activity assay, cells were allowed to adhere to PBS treated or fibronectin coated wells in the presence or absence of Ang-1. As revealed in S5 Fig, the combination of the two ligands leads to a significant increase in adhesion compared to the PBS control, which is not achieved by either Ang-1 or fibronectin alone. Importantly, the requirement for integrin is clear, as endothelial cells silenced for α_5_ no longer demonstrate a similar increase in adhesion under the same experimental conditions. Collectively, our data suggests a cooperative signaling event coordinated by the Tie2 and integrin α_5_ß_1_ signaling axis that promotes rapid cellular adhesion and activation of the MAPK pathway, a response that was not seen with either ligand alone.

## Discussion

The phenotypes of the integrin α_V_ and α_5_ null mice overlap substantially with those of the Tie2 and Ang-1 knockouts, suggesting that the two cooperate *in vivo*. Yet, compensation within the integrin family has prompted conflicting reports of their role in Tie signaling. Thus, to clarify the role of Tie/integrin receptor function, we utilized a combination of biochemical assays with purified protein components in conjunction with more complex cellular experiments. We reveal that the Tie receptors (both Tie1 and Tie2) constitutively associate with the endothelial integrins α_V_ß_3_ and α_5_ß_1_. Although the Tie2/α_V_ß_3_ complex has been loosely characterized previously, this is the first demonstration of a cell membrane partner for Tie1, aside from the recently described Tie1/Tie2 complex [11]. It is tempting to speculate that the ability of Tie1 to bind integrin receptors may provide an attractive explanation for the phenotype of the Tie1 null mouse, which perishes primarily from edema and hemorrhaging. Specifically, we would hypothesize that Tie1 is tethered to the basement membrane through interactions with the integrin molecules at sites of endothelial quiescence, providing additional stability to the endothelial cell. This would explain Tie1’s important role in endothelial integrity and response to alterations in shear stress [44–46]. The novel finding that Tie1 can couple with integrin molecules could, therefore, have significant implications for future therapies.

We further demonstrate that Tie/integrin recognition occurs readily in the presence or absence of the Tie2 ligands Ang-1 or Ang-2, and occurs through their respective extracellular domains, specifically within the three Ig and three EGF domains of the Tie’s. Furthermore, we reveal that Tie2/integrin interactions, but not those of Tie1/integrin, are specifically regulated by the ECM component fibronectin. However, Tie1 and Tie2 interactions with the integrins are mutually exclusive, suggesting that under certain conditions, integrin-Tie recognition may displace the inhibitory co-receptor Tie1 from the Tie1/Tie2 complex. Indeed, a dynamic equilibrium on the endothelial cell surface may dictate the formation of discrete membrane microdomains containing distinct Tie2 signaling complexes. Despite our detailed analysis with α_V_ß_3_ and α_5_ß_1_ integrins and fibronectin, it remains unclear if additional integrin heterodimers or ECM components also associate with Tie signaling proteins. Therefore, it is interesting to speculate that the potential for different integrin homodimers to associate with Tie receptors, would also enable distinct ECM components to selectively modulate specific signaling cascades. Thus, in light of the observation that only Tie2-integrin recognition is sensitive to, and significantly strengthened by, the integrin ligand fibronectin, one might predict that ECM components within the basal membrane, or in the nascent ECM observed by migratory endothelial cells during angiogenesis, for example, would greatly influence the equilibrium of Tie2 found in complex with either Tie1 or integrins. In this regard, it should be noted that several aspects of our complex mirror the well-characterized VEGFR2 interaction with integrin molecules, which is stabilized by the α_V_ß_3_ ligand, vitronectin. Furthermore, both Tie2 and VEGFR2 receptors display enhanced signaling potential in the presence of both integrins and matrix-associated Ang-1 or VEGF, respectively [21–25]. Mechanistically, the altered signaling characteristics may be a reflection of integrin-induced localization and recruitment of downstream signaling effectors.

Interestingly, previous studies concluded that Tie2 localization within the endothelial cell plasma membrane is critical for determining its unique signaling characteristics [47,48]. For example, in confluent endothelial cells in culture, Tie2 primarily promotes cell survival and vessel quiescence through Akt activation. Alternatively, Tie2 activation within sparse cells, or those lacking significant cell-cell contacts, predominantly enhances cellular proliferation and migration through stimulation of the MAPK signaling cascade. Though the exact molecular mechanisms behind this phenomenon were previously unknown, these observations can be collectively explained by our findings.

We also analyzed the binding of purified angiopoietin ligands to the integrins α_V_ß_3_ and α_5_ß_1_. The angiopoietins have been shown to have biological effects on cell types other than endothelial cells including breast cancer cells, cardiomyocytes, and neurons [34–36, 38, 40]. Ang-1 was shown to impact cardiomyocytes and neurons, cell types that lack the primary angiopoietin receptor Tie2, while Ang-2 was demonstrated to have metastatic effects in breast cancer cells through ß_1_ integrin. The ability of the angiopoietins to interact with another ubiquitous receptor type significantly increases the scope of this ligands function. Both Angiopoietin-1 and -2 have been previously implicated as potential binding partners, however in our assay, the receptor binding domain of Ang-1, but not that of Ang-2, binds directly to the integrin molecules α_V_ß_3_ and α_5_ß_1_ in the absence of other protein components using pure protein IPs and gel filtration analysis. Interestingly, Ang-1 lacks a canonical integrin recognition RGD sequence suggesting that a novel interface is likely employed. In agreement, we find that fibronectin binding does not compete, nor facilitate Ang-1/integrin association.

We were surprised to observe that Ang-1/integrin association did not appear to significantly stabilize the Tie2 / integrin complex (Figs 1A and 3B), though it also didn’t interfere with Tie2 binding. The amino-terminus of each integrin subunit mediates heterodimer formation to create the “head” domain responsible for association with the extracellular matrix component ligand. Functional regulation of the integrins is controlled by large conformational changes in the extracellular domain, which may form either a significantly bent inactive conformation in which 2000Å^2^ of surface area is buried, or a significantly more solvent exposed and extended conformation. The extended conformation may itself also exist with either a “closed” head conformation similar to that in the inactive bent conformation, or an “open” head conformation that allows for ligand ligation [49–52]. Since integrin-ligand recognition induces extension of the receptor, and fibronectin binding regulates Tie2/integrin association, we might predict that Tie2 preferentially recognizes the extended and activated conformation. However, in light of the observation that Tie1 and Tie2 compete for binding, and Tie1/integrin association is not regulated by ligands, it would appear more likely that fibronectin stabilizes the Tie2/integrin complex by actively binding both integrin and Tie2. Our hypothesis is supported by the observation that recombinant fibronectin spanning only the 9th and 10th fibronectin type-III repeats efficiently binds α_5_ß_1_ and induces receptor extension, but unlike the full-length protein, does not enhance Tie2/integrin association.

Finally, as mentioned earlier, several previous studies have documented specific interactions between Ang-2 and various integrin heterodimers [34–36, 38, 40]. Unfortunately, we were not able to establish direct binding between Ang-2 and either α_V_ß_3_ or α_5_ß_1_ in our pure protein assays. However, it should be noted that for our binding assays, we utilized the fibrinogen-like receptor-binding domain of the angiopoietin ligands, Ang-1 and -2, which are significantly easier to express and purify than the full-length ligands. Thus, it should be noted that for Ang-2, we are unable to rule out the possibility that specific interactions with integrin receptors may occur outside the fibrinogen-like domain. In this regard, Hakanpaa et al. recently reported an interaction between the amino-terminal region of Ang-2 and the integrin ß_1_ subunit [53]. Similarly, studies in HUVEC’s show an interaction between Ang-2 and α_V_ß_3,_ α_5_ß_1_, and α_V_ß_5_, utilizing the full-length protein [10]. Thus, while Ang-1 uses the fibrinogen-like domain for integrin association, Ang-2 may alternatively utilize the amino-terminal coiled-coil domain. Additional studies will be required to assess if the Ang-2 interactions are also direct, and indeed utilize the amino-terminal coiled-coil domain as proposed.

**Fig 6:**
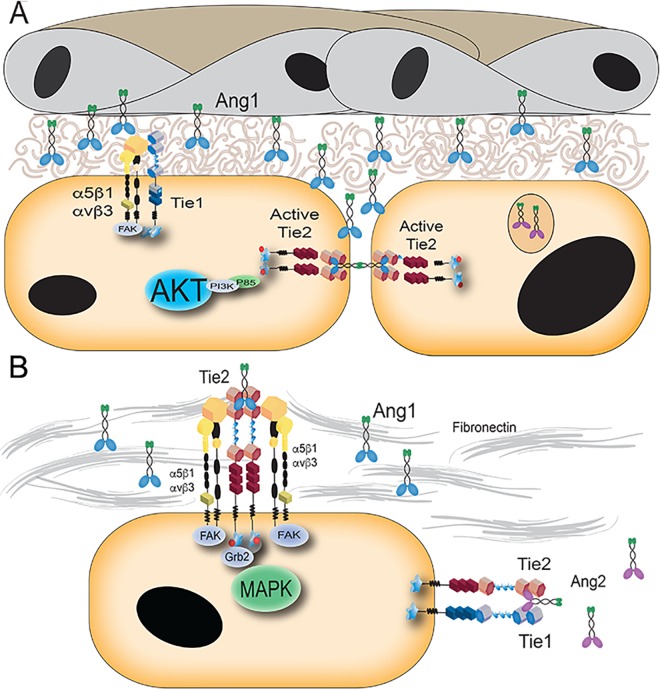
Proposed model of Integrin Tie2 cooperative signaling in endothelial cell regulation. (A) Based on previous studies, it is known that Ang-1 clusters Tie2 at cell-cell junctions in endothelial cells while providing pro-survival signals through the AKT and Survivin signal transduction pathway [30,46,47]. Alternatively, Tie1 may associate with integrins, including either α_V_ß_3_ or α_5_ß_1_, which are preferentially localized at the cell interface with the basement membrane containing extracellular matrix components. A link between Tie1 and the extracellular matrix could explain a role for Tie1 in vessel integrity and sheer stress that has previously been described [43–45]. (B) When Tie2 is not at junctions, it is likely capable of associating with integrins. Activation of integrin-associated Tie2 via Ang-1 and fibronectin may enrich signaling through MAPK to further facilitate endothelial cell migration and proliferation.

To incorporate the physical interactions and signaling data presented, in addition to previously reported data highlighting the spatial and temporal regulation of Tie2, we propose the following model (Fig 6). In a quiescent endothelium, Tie2 primarily localizes to cell-cell contacts where Ang-1 can act as a bridge to associate Tie2 molecules in *trans* and increase the stability of endothelial cell junctions, as shown previously [47,48]. From these Tie2 clusters at cell junctions, Ang-1 promotes pro-survival signaling through Akt and its downstream effectors. Alternatively, Tie1 is known to play an important role in shear stress and is up-regulated at vascular branch-points where there is increased force exerted on the endothelial cells by turbulence and non-laminar flow. Thus, at cell-extracellular matrix contact points in the resting endothelium, Tie1 and integrin receptors likely form a complex that strengthens the integrity of the wall to the basement membrane. Additionally, Tie1 knockout mice exhibit increased hemorrhaging and edema, a phenotype that has not been well understood in the field. Cross-talk between integrins and Tie1 could explain this distinct phenotype due to decreased adhesion to the surrounding extracellular matrix.

During vessel branching, post stimulation with VEGF and Ang-2, endothelial cells detach, reduce cell junction contacts and begin to migrate. Under these conditions, we postulate that Tie2 is redistributed following dissolution of the endothelial cell-cell contacts, where it is able to associate with the integrins contacting extracellular matrix. With Ang-1 attached to extracellular matrix components through its free cysteine, Tie2 and the integrins coordinately signal through MAPK to promote migration and proliferation of branching vascular endothelial cells.

## Materials and Methods

### Cell culture and manipulation

HEK293, HEK293t, and EA.hy926 cells (ATCC) were cultured in Dulbecco’s Modified Eagles Medium (Invitrogen) with 10% Fetal Bovine Serum (Gemini Bio-Products), 100 U/mL Penicillin and 100 μg/mL Streptomycin (Gibco Life Technologies). Transfections were performed when cells reached 80–90% confluence using Lipofectamine 2000 (Invitrogen) according to manufacturers instructions either in 6-well dishes for immunoprecipitations (Greiner) or 35 mm glass bottom culture dishes for imaging analysis (MatTek). Equimolar concentrations of plasmid vectors were used for transfections when necessary. Telomerase-immortalized microvascular endothelial (TIME) cells (ATCC) were grown in Endothelial Basal Media-2 (EBM-2) supplemented with the Endothelial Growth Media-2 (EGM-2) Microvascular (MV) SingleQuot kits (Lonza).

### Lentiviral-mediated protein silencing

HEK293t cells were transfected with the pGipz control vector or the pGipz shRNAmir containing plasmids targeting α_5_ (clone number 133433) or α_V_ (clone number 133468) using the Arrest-In lipid transfection reagent (ThermoScientific) in serum and antibiotic free media. After three hours of incubation, DMEM containing 10% FBS, 100 U/mL Penicillin and 100 μg/mL Streptomycin was added. After an additional 48 hours the virus was harvested from the media following clarification by centrifugation at 3000 rpm for 20 minutes. The viral supernatant was directly added to ~80% confluent TIME cells and incubated for 48 hours. Transduced cells were selected using 1 μg/mL of puromycin (Invitrogen) until the cells were 100% GFP positive. Protein levels were checked by western blot using antibodies to α_5_ (1: 1000 dilution, Cat. 4705, Cell Signaling), α_v_ (1:1000 dilution, Cat. 4711, Cell Signaling) and Actin proteins (1:5000 dilution, A2228, Sigma Aldrich).

### Co-immunoprecipitation

6-well dishes (Greiner) were treated overnight with either PBS (Invitrogen) or 5 μg/mL fibronectin (R&D systems) diluted in PBS as indicated. For immunoprecipitations of endogenous protein, EA.hy 926 cells were grown on treated dishes until confluent, starved in serum free media for 4 hours and treated with vehicle, Ang-1 or Ang-2 (R&D) at 500 ng/mL for 30 minutes at 37 degrees Celsius. Cells were rinsed with cold PBS and scraped from the plates in 1% NP-40 lysis buffer (20 mM Hepes pH 7.4, 150 mM NaCl, 1 mM EDTA, and 1% NP-40 supplemented with PhosStop and Complete (Roche)). Lysates were incubated on ice for 10 minutes and spun at 15k rpm for 5 minutes. Clarified lysate (0.5 mg) was incubated with 25 μL of Protein-A beads in 20 mM Hepes pH 7.4, 150 mM NaCl, 0.1% TritonX-100 buffer (HBST) for 1 hour alone. The supernatant was transferred to fresh beads and incubated with 2 μg of Tie2 C20 antibody (sc-324 Santa Cruz) or control overnight at 4 degrees Celsius while rotating. Beads were washed twice with HBST and once with HBS before addition of 1x SDS dyes and resolution on a 10% SDS polyacrylamide gel. Protein was transferred to nitrocellulose and immunoblotted using an anti-α_V_ antibody (1:1000 dilution, Cat. 4711, Cell Signaling).

HEK293 transfected cell lysates were immunoprecipitated in a similar manner with 1 mg of lysate, 2 μg Flag antibody (Syd Labs) or IgG control, and 25 μL of Protein-A beads overnight. Anti-GFP (1:5000 dilution, Cat. MMS-118R, Covance), anti-HA (1:5000 dilution) (12CA5), anti-myc (1:5000 dilution) (9E10) and anti-Flag antibodies were used for immunoblotting.

Pure protein immunoprecipitations were incubated overnight with all proteins at 2.5 μg/mL unless specifically indicated in the figure (α_5_ß_1_ = 12.5nM, Tie2(1–4)-Fc = 33nM, Tie1(1–4)-Fc = 33nM, Tie2 full ectodomain = 24nM, Ang1-RBD-Fc = 50nM, Ang2-RBD-Fc = 50nM) in 1 mL of HBST buffer and 25 μL of ProteinA beads. Beads were washed as previously described and protein was separated via 10% SDS-PAGE run at a constant 200V. Protein was transferred to polyvinylidene fluoride (PVDF) at 25V, in some cases visualized with Ponceau S stain, blocked for one hour in 5% (w/v) evaporated milk in TBST, and immunoblotted overnight with antibodies against Tie2 (AB33) (1:1000 dilution, Cat. 4224, Cell Signaling), ß_1_ (1:1000 dilution, Cat. 4706, Cell Signaling), or ß_3_ (1:1000 dilution, Cat. 4702, Cell Signaling).

### Protein purification

Integrin expression constructs were reproduced based on the work of Mehta et al and Xiong et al [52,54]. Human ß_1_ (residues 1–728) (TrueORF gold cDNA construct RC203818 (Origene Technologies, inc.)) and human ß_3_ (residues 1–718) (TrueORF gold cDNA construct RC201151 (Origene Technologies, inc.)) were amplified and integrated into a pCDNA3.1(+)-Fc neo containing backbone by overlap PCR introducing a thrombin cleavage site at the C-terminus of the protein and before an Fc fragment. Human α_5_ (residues 1–995) and human α_V_ (residues 1–987) were similarly cloned into a pCDNA3.1(+)-Fc hygromycin backbone. Single cell cloned stable lines for the Fc-tagged α integrin constructs were created in HEK293 cells using 150 mg/mL Hygromycin B (Invitrogen) for selection. The appropriate ß-Fc construct was then transfected into these stable cells using Lipofectamine 2000 and selected for using 0.5 mg/mL G418 sulfate (Gemini Bio-Products). The angiopoietin receptor binding domains, Tie1 (1–4) (residues 1–445) and Tie2 (1–4) (residues 1–452) were purified as previously described [55]. Tie2 Full ectodomain (1–729) was similarly cloned with a TEV protease site replacing the Thrombin cleavage site. Briefly, stable cell lines were grown in roller-bottle culture and secreted protein was purified from the media using a packed Protein-A sepharose column on an Akta Purifier FPLC system (GE). Pure Fc-tagged protein was then cleaved overnight at room temperature using 2 U/mg Thrombin (Novagen) or a 1:300 ratio of purified TEV to target protein and separated from the Fc tag using a HiTrap Protein- A HP column (GE Healthcare). Any minor contaminates were removed via size exclusion chromatography (Superdex 200 10/300) (GE Healthcare).

### Complex formation by gel filtration chromatography

5μM purified α_5_ß_1_ was incubated with 10μM purified Ang1-RBD for 1 hour on ice. Excess ligand was used to force all integrin protein into a complex. 250μM DTSSP was added to the complex and incubated on ice for 2 hours. The reaction was quenched with 50mM Tris pH 8 for 15 minutes before filtering and running on a Superdex200 10/300 column. The individual protein runs were treated in a similar manner.

### Surface plasmon resonance experiments

The binding interactions between Ties and α_5_ß_1_ were analyzed in real-time by surface plasmon resonance (SPR) spectroscopy using ProteOn XPR36 instrument (Bio-Rad, CA, USA). ProteOn GLC sensor chip was air initialized and PBST (PBS x1, 0.005% Tween) buffer was flushed through the instrument prior to binding measurements. Recombinant human Tie1 (rhTie1) extracellular domain and recombinant human Tie2 (rhTie2) were immobilized on the surface of a GLC sensor chip (Bio-Rad) using the amine coupling reagents sulfo-NHS (0.1M N-hydroxysuccinimide) and EDC (0.4M 1-ethyl-3-(3-dimethylaminopropyl) -carbodiimide, Bio-Rad). rhTie1 and rhTie2 (2 μg both) in 10 mM sodium acetate pH 5.0 were flowed over the activated surfaces of the GLC sensor chip channels at a flow rate of 30 μL/min until the target immobilization levels of 4750 RU and 6400 RU respectively were reached. BSA (3 μg) in 10 mM sodium acetate pH 4.5 was then flowed over additional activated surfaces of a control GLC sensor chip channel at a flow rate of 30 μL/min until the target immobilization levels of 4946 RU was reached. After protein immobilization, chip surface was treated with 1M ethanolamine-HCl at pH 8.5 in order to deactivate the excess of reactive esters. All binding experiments were performed at 25 degrees Celsius in degassed integrin binding buffer (IBB, 20 mM Tris, 100 mM NaCl, 1 mM MnCl_2_, 2 mM CaCl_2_ pH 7.5). α_5_ß_1_ integrin at a concentration of 3 μM was flowed over the surface-immobilized rhTie1 and rhTie2 at a flow rate of 60 μL/min for 3 minutes and the binding interactions were monitored. Following association, the dissociations of the various ligand-receptor complexes were monitored for 5 minutes. Each sensogram run was normalized by subtracting the BSA channel run and the blank analyte run (at zero concentration).

### Molecular imaging

Live cell FRET assays were based on earlier work [11]. Images were taken 24–48 hours post transfection of HEK293 cells using a Zeiss Observer Z.1 incubated wide- field microscope with a 63X glycerin Plan Neofluar DIC optic with a 1.3 NA for sensitized emission data. The CFP variant, mTurquoise, (kind gift of J. Goedhart) with a higher intensity and single exponential decay was used as a donor fluorophore in our sensitized emission and FLIM FRET based proximity experiments [56]. mTurquoise was excited by LED (Colibri) at 430 nm through a filter cube containing a dichroic beamsplitter for eCFP and eYFP (Chroma 89002bs) and either an emission filter specific for CFP control measurements (ET470/24m) or YFP (ET535/30m) for FRET measurements. YFP variants were excited at 505 nm by LED and imaged through the YFP beamsplitter and emission filter described above for direct excitation controls. All sensitized emission measurements were obtained using the same exposure time for all images within an experiment, generally between 500–800 ms. Sensitized emission data was analyzed using the PFRET ImageJ plugin in addition to the contrast EP plugin for final FRET image acquisition [57]. Statistical analysis and graphing was performed in JMP Pro 11. For fluorescence lifetime measurements, a Zeiss 510 Meta with a mode locked 80 MHz Ti:Sapphire laser and Becker and Hickl TCSPC fluorescence lifetime instrument was used. FLIM-FRET measurements were obtained using the Becker and Hickl SPC- 130 software in FiFo mode with 10% laser power. Decay matrix calculation and analysis was performed in SPC-Image.

Acceptor photobleaching experiments were conducted on transfected cells as described above. Briefly, imaging was conducted on a Zeiss 710 with a 63x oil immersion objective using 1Au. All images were taken using constant laser power between all images within one experiment. Similarly, the gain was set and held constant within one experiment. Regions of interest within the field of view were photobleached using 100% 514nm laser power for two complete scans. Images of both donor (440 nm laser) and acceptor (514 nm laser) channels were collected pre and post photobleaching. Bleached samples depict the percent change in donor signal within an ROI following at least 50% bleaching of the acceptor molecule. Unbleached samples are internal controls measuring the percent change in donor fluorophore intensity ROI values in regions of the field of view that were not subjected to intense acceptor laser scanning. Unbleached measurements were collected to control for cell movement or variation of donor intensity levels not induced by acceptor bleaching. Images were background corrected and analyzed using FIJI and the FRETcalc plugin v5.0 to measure changes in donor intensity within regions of interest [58]. Statistical analysis and graphing was performed in JMP Pro 11.

### Tie2 Activation Assays

6-well plates were treated with either PBS or 10 μg/mL fibronectin overnight at 4 degrees Celsius. TIME cells were trypsinized (Invitrogen), quenched with full media, and spun at 1000 rpm for five minutes. Pelleted cells were resuspended in serum free media and incubated at 37 degrees Celsius with shaking for 1 hour. Serum free media was added to either PBS or fibronectin treated dishes, with vehicle, Ang-1 at 500 ng/mL or Ang-2 at 500ng/mL. Suspended cells were distributed equally in the wells and allowed to incubate at 37 degrees Celsius for 15 minutes (Fig 5), 30 minutes or 1 hour (S5C Fig). Cells were washed in cold PBS and lysed as described previously. 50 μg of cell lysate was resolved via SDS-PAGE and immunoblotted for total Akt (1:1000 dilution, Cat. 9272, Cell Signaling), MAPK (1:1000 dilution, Cat. 4695, Cell Signaling), and FAK (1:1000 dilution, Cat. 3285, Cell Signaling) or specific phosphorylated residues (1:1000 dilution, threonine 308 of Akt (Cat. 4056), threonine 202 and tyrosine 204 of MAPK (Cat. 4695), or tyrosine 397 of FAK (Cat. 3283, Cell Signaling). Densitometry was performed using gel analysis software (Fiji) and statistically analyzed with JMP Pro 11.

### Adhesion Assay

96-well plates were coated with either PBS or 10 μg/mL fibronectin overnight at 4 degrees Celsius. TIME cells (shControl or shα_5_) were trypsinized, quenched with full media, and spun at 300*g for three minutes. Pelleted cells were resuspended in serum free media and incubated at 37 degrees Celsius with shaking for 1 hour. 100μL of serum free media containing 10,000 cells with vehicle or Ang-1 at 500 ng/mL was added to either PBS or fibronectin treated dishes. Cells were allowed to adhere in a 37 degree Celsius inbubator for 30 minutes. Wells were washed twice with PBS and fixed with cold methanol for 10 minutes. After fixation, wells were washed three times with PBS and stained with 0.5% Crystal Violet in 20% Methanol. Adherent cells were counted under 5x magnification.

## Supporting Information

S1 Fig Coomassie stained SDS-Page gels of purified integrin, angiopoietins, and Tie receptors. (TIF)

S2 Fig Pure Protein Immunoprecipitation of Tie1 and integrin α_5_ß_1_ in the presence of fibronectin. Fibronectin (5μg/mL) does not influence the association of Tie1 (33nM) and integrin α_5_ß_1_ (10nM). Full-length and recombinant (9–10) fibronectin were tested for their ability to modulate Tie1/integrin interactions. Unlike Tie2, association between Tie1 and integrins is not sensitive to fibronectin. The same procedure used in Fig 2 was employed here.
(TIF)

S3 Fig FRET Analysis of α_5_ß_1_/Tie2 and α_5_ß_1_/Tie1 interactions including all controls. (A) FLIM-FRET measurements were conducted on the same transfections used for sensitized emission measurements. Lifetime values described are the peak values of the fast component after the slow components were fixed to donor alone control. Tie2-mTQ alone: 3.09 ns; Tie2-PlexinA1-YFP: 3.00 ns; Tie2-mTQ/Tie1-sYFP2: 2.39 ns; Tie2- mTQ/α_5_-YFP/ß_1_ -mCherry: 2.55 ns; Tie2- mTQ/α_5_-YFP/ß_1_-mCherry in the presence of 5 μg/mL fibronectin: 2.5 ns. Images and decay matrix analysis was performed in SPCImage. (B) Jablonski diagram describing lifetime values as related to Fluorescence Resonance Energy Transfer. (C) Graphical representation of acceptor photobleaching FRET experiments between Tie1-mTQ and α_5_-YFP, as well as a negative control (PlexinA1-YFP/Tie2-mTQ) and positive control (Tie2-mTQ/ Tie1-sYFP2). Bleached samples depict the percent change in donor signal following at least 50% bleaching of the acceptor molecule. Unbleached samples are internal controls measuring the percent change in donor fluorophore intensity ROI values in regions of the field of view that were not subjected to intense acceptor laser scanning to control for cell movement or variation not induced by acceptor bleaching. The white arrow indicates one bleached ROI while the red arrow indicates a control ROI outside of the bleached region. Tie1 and α_5_ß_1_ show significant FRET (11.0%+/- 1.3%) above the negative control value (3.4% +/- 1.8%) indicating that Tie1 and α_5_ß_1_ interact directly at the cell surface in living cells. The positive control value of Tie1-sYFP2 and Tie2-mTQ using this method is 16.8% +/- 1.1%. Error bars represent the standard error of n>3 over three independent experiments. * represents student t-test values p<0.01. (B) Images representing one sample of the positive control Tie1-sYFP2 and Tie2-mTQ.
(TIF)

S4 Fig Downstream signaling mediators of Tie and integrin activation followed over time. (A) A time-course of Akt and FAK activation following plating on mock-treated, or fibronectin treated dishes. Although Akt activation remains stable across all time points tested, levels of p-FAK consistently increases with fibronectin treatment. (B) Vehicle, 500 ng/mL Ang-1, or 500 ng/mL Ang-2 stimulation of control TIME cells or TIME cells knocked down for α_5_ (knockdown verified in Fig 5E). Akt T308 phosphorylation levels and total Akt levels were monitored by western blot. Decreasing the level of α_5_ protein did not significantly affect Ang-1 initiated Akt signaling. (C) Serum starved telomerase-immortalized endothelial cells (TIMEs) were plated on control treated or fibronectin treated dishes with vehicle stimulation, 500 ng/mL Ang-1, or 500 ng/mL Ang-2. MAPK activation was monitored over 60 minutes following plating. At 30 minutes post-plating, fibronectin increases p-MAPK levels; at 15 minutes, only the combination of fibronectin and Ang-1 significantly increase p-MAPK levels.
(TIF)

S5 Fig Fibronectin and Ang-1 increase adhesion in serum starved Telomerase Immortalized Microvascular Endothelial Cells. shControl and shα_5_ TIME cells were serum starved for 1 hour in suspension before plating on tissue culture treated 96 well plates for 30 minutes at 37 degrees. The combination of 10 μg/mL fibronectin and 500 ng/mL Ang-1 significantly increased adhesion over PBS treated wells under these conditions (Student’s t-test; p<0.05). Conditions were completed in triplicate with at least two independent experiments.
(TIF)

S6 Fig Unmanipulated images for Fig 1. (TIF)

S7 Fig Unmanipulated images for Fig 2. (TIF)

S8 Fig Unmanipulated images for Fig 3. (TIF)

S9 Fig Unmanipulated images for Fig 5. (TIF)

S10 Fig Unmanipulated images for supplemental images. (TIF)
