# Correction: Constitutive Association of Tie1 and Tie2 with Endothelial Integrins is Functionally Modulated by Angiopoietin-1 and Fibronectin

**DOI:** 10.1371/journal.pone.0179059

**Published:** 2017-05-31

**Authors:** Annamarie C. Dalton, Tomer Shlamkovitch, Niv Papo, William A. Barton

In Fig 1, the control panel at the bottom of panel C is incorrectly duplicated. Please see the correct Fig 1 and its caption below.

**Fig 1:**
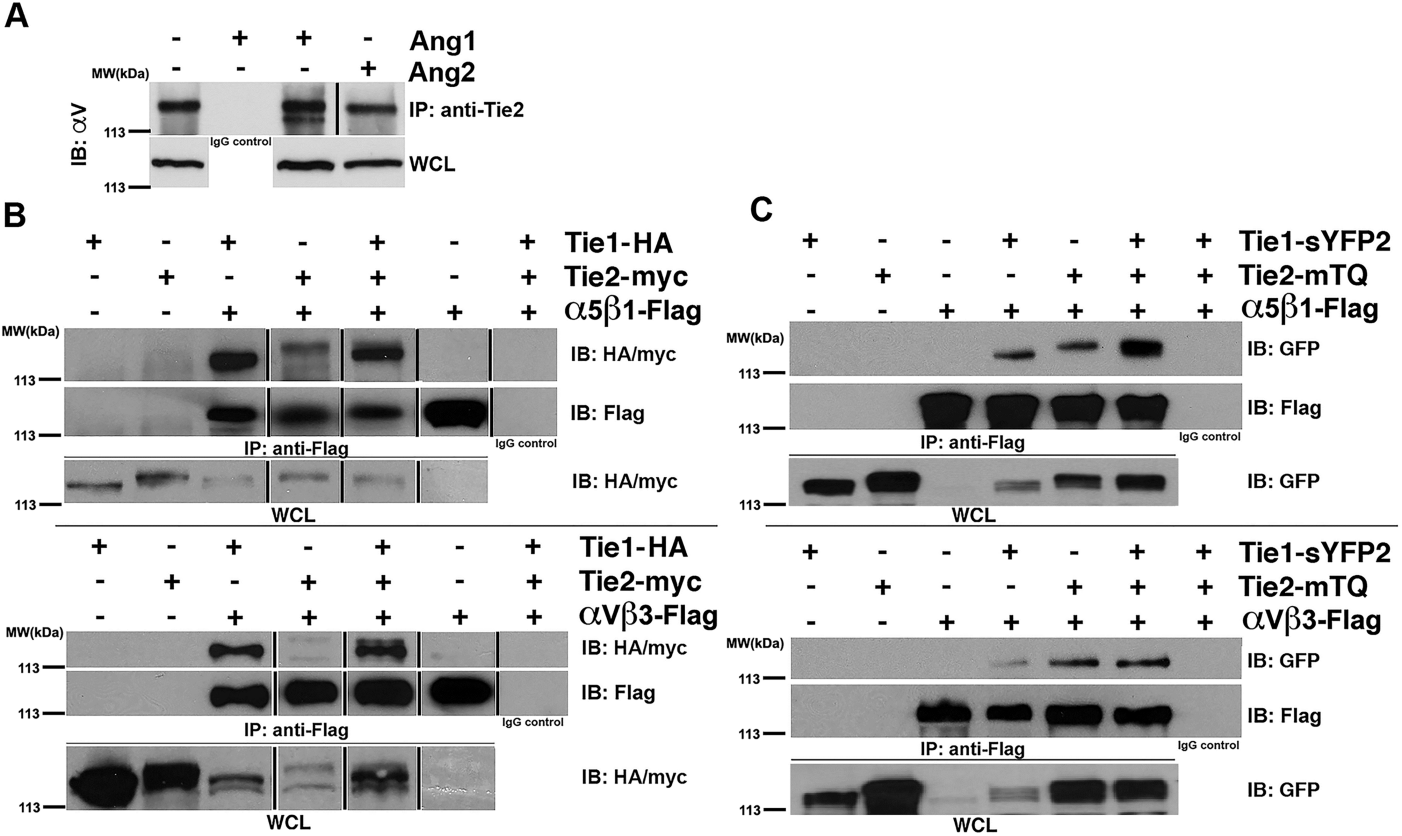
Tie1 and Tie2 interact with the endothelial Integrins αVß3 and α5ß1 through their extracellular domains. (A) Co-immunoprecipitation of endogenous αV with Tie receptor in Ea.hy926 endothelial cells. Cells were serum starved and then treated with vehicle control (PBS), 500 ng/mL rhAng-1 or 500 ng/mL rhAng-2 for 30 minutes at 37 degrees Celsius prior to harvest. αV co-precipitated under all stimulation conditions tested when lysates were incubated overnight with anti-Tie2 antibody, but not when incubated with a non-specific IgG. (B-C) Co-immunoprecipitation of αVß3 and α5ß1 with Tie receptors from transiently transfected HEK293 cells. (B) Full-length integrins α5ß1 (top panel) or αVß3 (bottom panel) were immunoprecipitated with anti-flag antibody, while co-precipitating HA-tagged Tie1 receptor or myc-tagged Tie2 receptor were detected by western blot using anti-HA or anti-myc antibodies, respectively. (C) Same as in (B) alterations in the transfected vectors. Here, Tie1 and Tie2 receptor fluorophore fusion constructs were used in which the intracellular tyrosine kinase domains were replaced with the GFP analogues sYFP2 and mTQ GFP variants respectively. Co-precipitating Tie1 and Tie2 fusion proteins were detected by western blot with anti-GFP antibodies indicating receptor association within the extracellular domains of the proteins (α5ß1- top panel and αVß3—bottom panel). WCL- Whole Cell Lysate. IP- Immunoprecipitation. IB: Immunoblotting.

In S6 Fig, the far right image of panel C appears incorrectly. Please see the correct S6 Fig and its caption below.

## Supporting information

S6 Fig Unmanipulated images for Fig 1. (TIF)
