# Supplementary figures and images for: Constitutive Association of Tie1 and Tie2 with Endothelial Integrins is Functionally Modulated by Angiopoietin-1 and Fibronectin
Source: PLoS One. 2016 Oct 3;11(10):e0163732. doi: 10.1371/journal.pone.0163732 (PMC5047623; doi:10.1371/journal.pone.0163732)

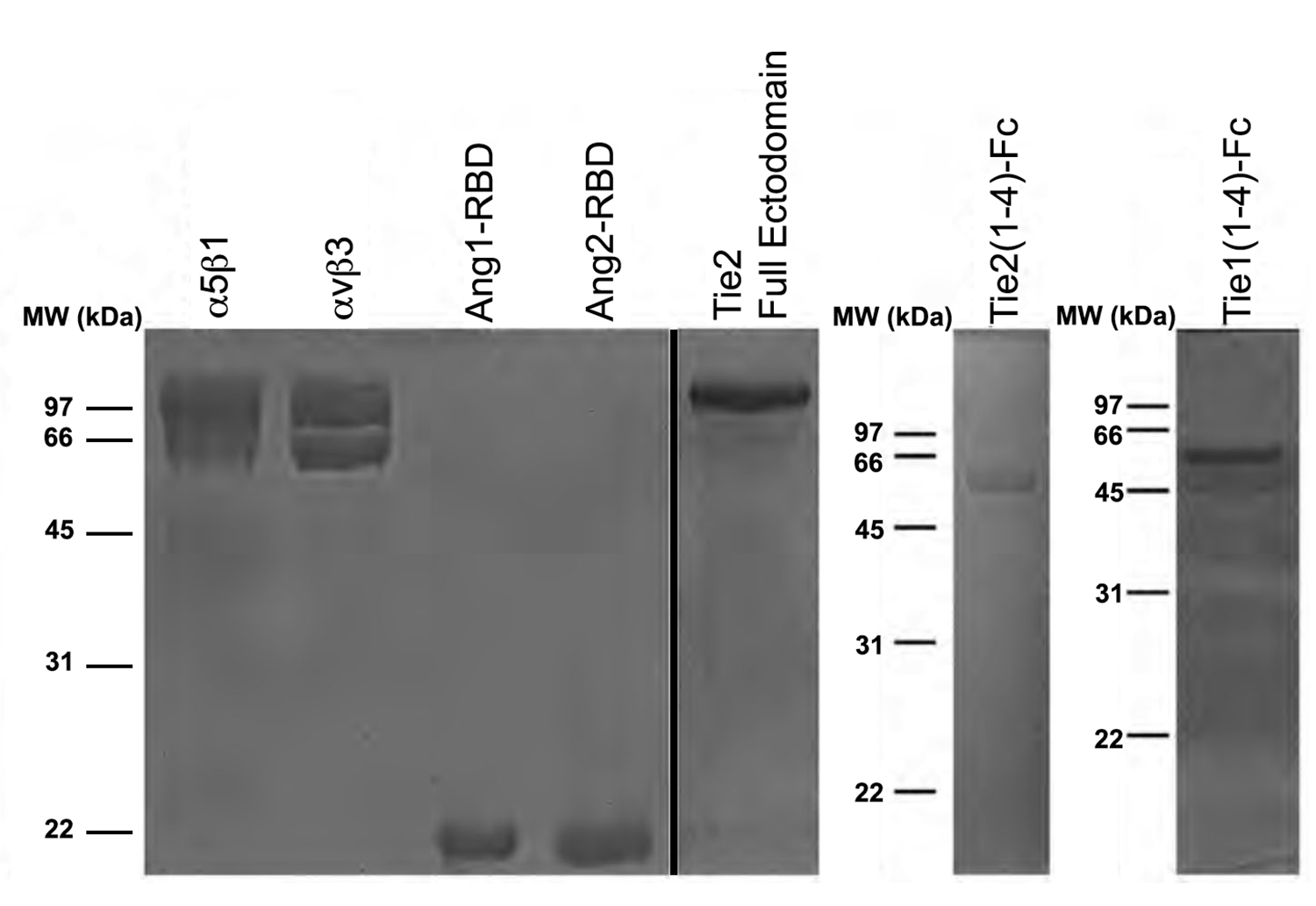

Supplement: S1 Fig — (TIF) [file pone.0163732.s001.tif]

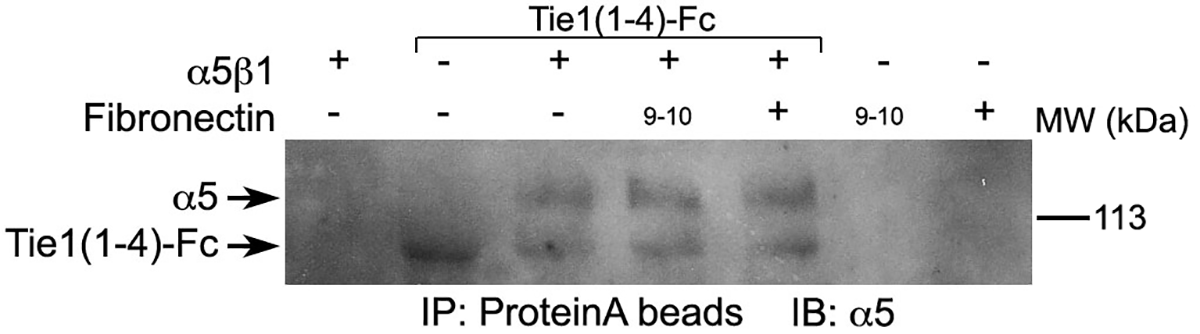

Supplement: S2 Fig — Fibronectin (5μg/mL) does not influence the association of Tie1 (33nM) and integrin α5ß1 (10nM). Full-length and recombinant (9–10) fibronectin were tested for their ability to modulate Tie1/integrin interactions. Unlike Tie2, association between Tie1 and integrins is not sensitive to fibronectin. The same procedure used in Fig 2 was employed here. (TIF) [file pone.0163732.s002.tif]

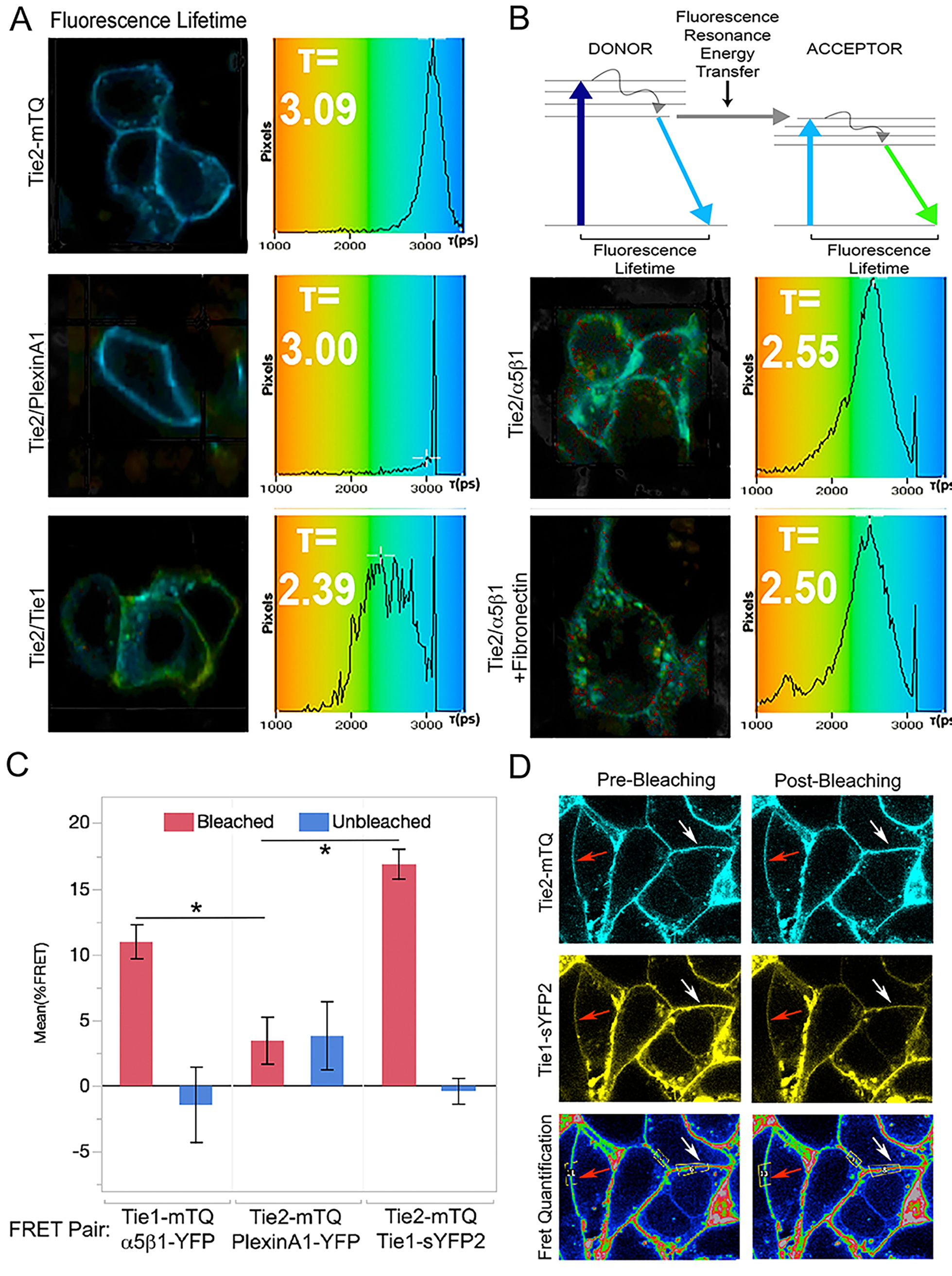

Supplement: S3 Fig — (A) FLIM-FRET measurements were conducted on the same transfections used for sensitized emission measurements. Lifetime values described are the peak values of the fast component after the slow components were fixed to donor alone control. Tie2-mTQ alone: 3.09 ns; Tie2-PlexinA1-YFP: 3.00 ns; Tie2-mTQ/Tie1-sYFP2: 2.39 ns; Tie2- mTQ/α5-YFP/ß1 -mCherry: 2.55 ns; Tie2- mTQ/α5-YFP/ß1-mCherry in the presence of 5 μg/mL fibronectin: 2.5 ns. Images and decay matrix analysis was performed in SPCImage. (B) Jablonski diagram describing lifetime values as related to Fluorescence Resonance Energy Transfer. (C) Graphical representation of acceptor photobleaching FRET experiments between Tie1-mTQ and α5-YFP, as well as a negative control (PlexinA1-YFP/Tie2-mTQ) and positive control (Tie2-mTQ/ Tie1-sYFP2). Bleached samples depict the percent change in donor signal following at least 50% bleaching of the acceptor molecule. Unbleached samples are internal controls measuring the percent change in donor fluorophore intensity ROI values in regions of the field of view that were not subjected to intense acceptor laser scanning to control for cell movement or variation not induced by acceptor bleaching. The white arrow indicates one bleached ROI while the red arrow indicates a control ROI outside of the bleached region. Tie1 and α5ß1 show significant FRET (11.0%+/- 1.3%) above the negative control value (3.4% +/- 1.8%) indicating that Tie1 and α5ß1 interact directly at the cell surface in living cells. The positive control value of Tie1-sYFP2 and Tie2-mTQ using this method is 16.8% +/- 1.1%. Error bars represent the standard error of n>3 over three independent experiments. * represents student t-test values p<0.01. (B) Images representing one sample of the positive control Tie1-sYFP2 and Tie2-mTQ. (TIF) [file pone.0163732.s003.tif]

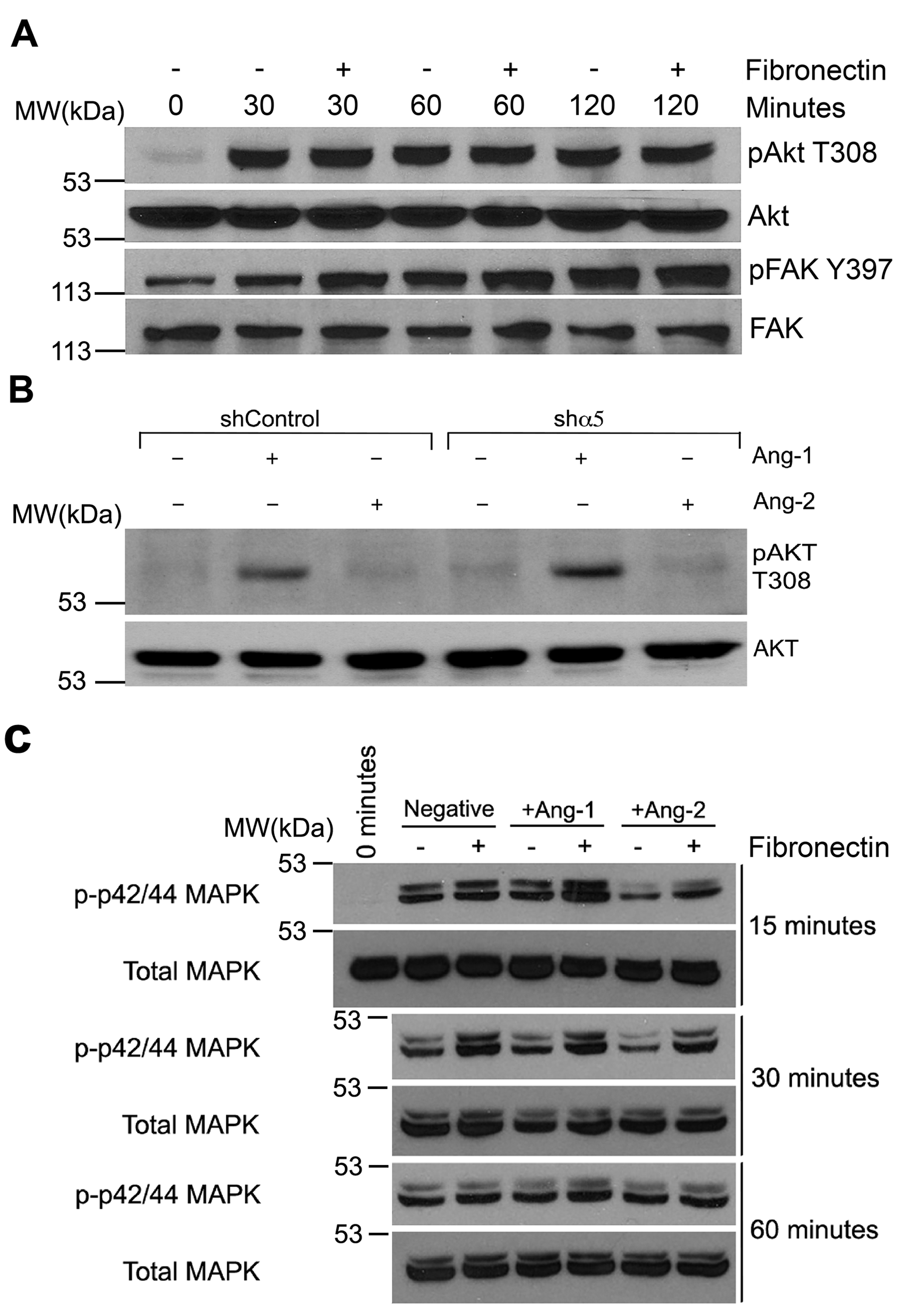

Supplement: S4 Fig — (A) A time-course of Akt and FAK activation following plating on mock-treated, or fibronectin treated dishes. Although Akt activation remains stable across all time points tested, levels of p-FAK consistently increases with fibronectin treatment. (B) Vehicle, 500 ng/mL Ang-1, or 500 ng/mL Ang-2 stimulation of control TIME cells or TIME cells knocked down for α5 (knockdown verified in Fig 5E). Akt T308 phosphorylation levels and total Akt levels were monitored by western blot. Decreasing the level of α5 protein did not significantly affect Ang-1 initiated Akt signaling. (C) Serum starved telomerase-immortalized endothelial cells (TIMEs) were plated on control treated or fibronectin treated dishes with vehicle stimulation, 500 ng/mL Ang-1, or 500 ng/mL Ang-2. MAPK activation was monitored over 60 minutes following plating. At 30 minutes post-plating, fibronectin increases p-MAPK levels; at 15 minutes, only the combination of fibronectin and Ang-1 significantly increase p-MAPK levels. (TIF) [file pone.0163732.s004.tif]

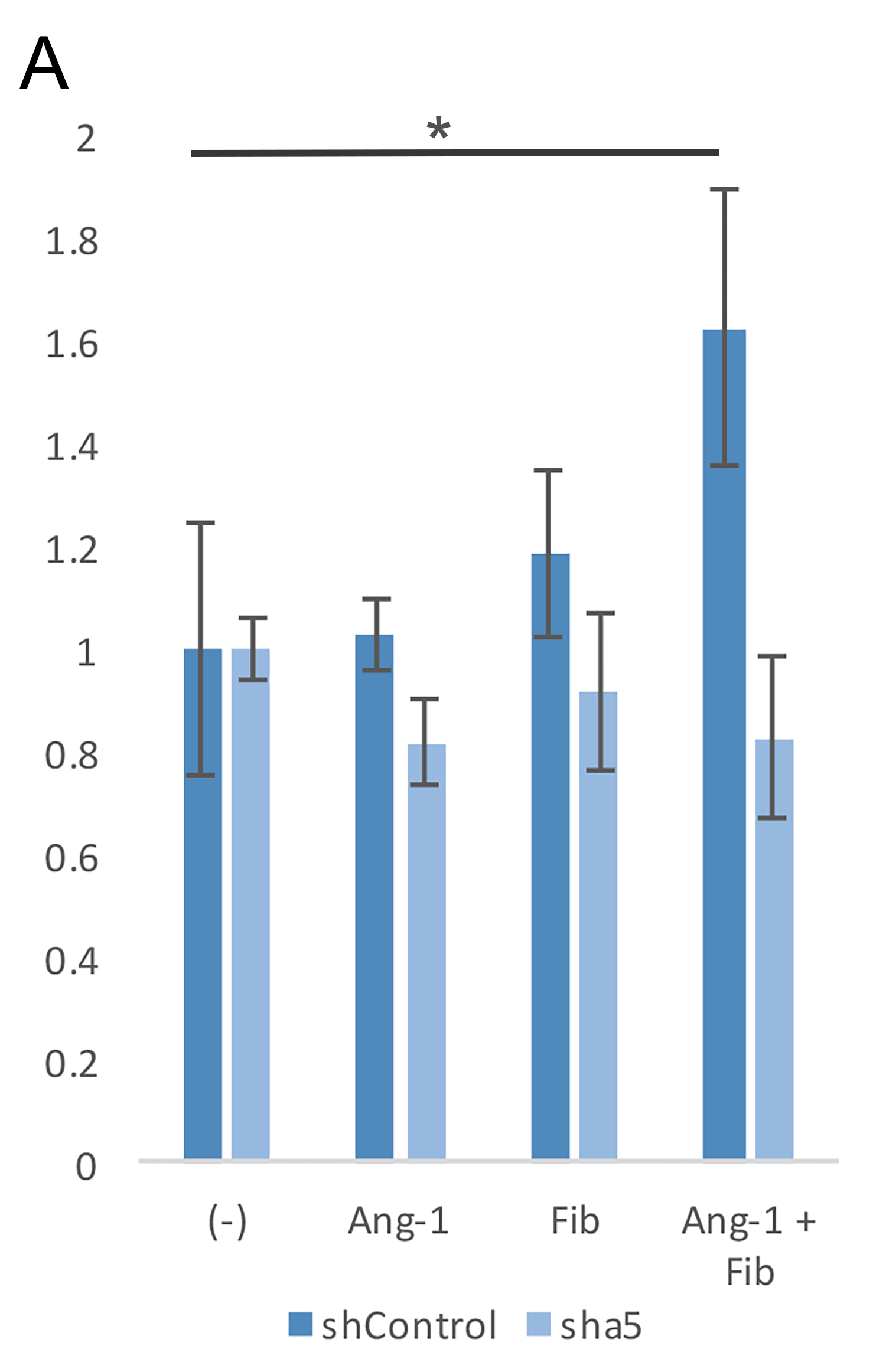

Supplement: S5 Fig — shControl and shα5 TIME cells were serum starved for 1 hour in suspension before plating on tissue culture treated 96 well plates for 30 minutes at 37 degrees. The combination of 10 μg/mL fibronectin and 500 ng/mL Ang-1 significantly increased adhesion over PBS treated wells under these conditions (Student’s t-test; p<0.05). Conditions were completed in triplicate with at least two independent experiments. (TIF) [file pone.0163732.s005.tif]

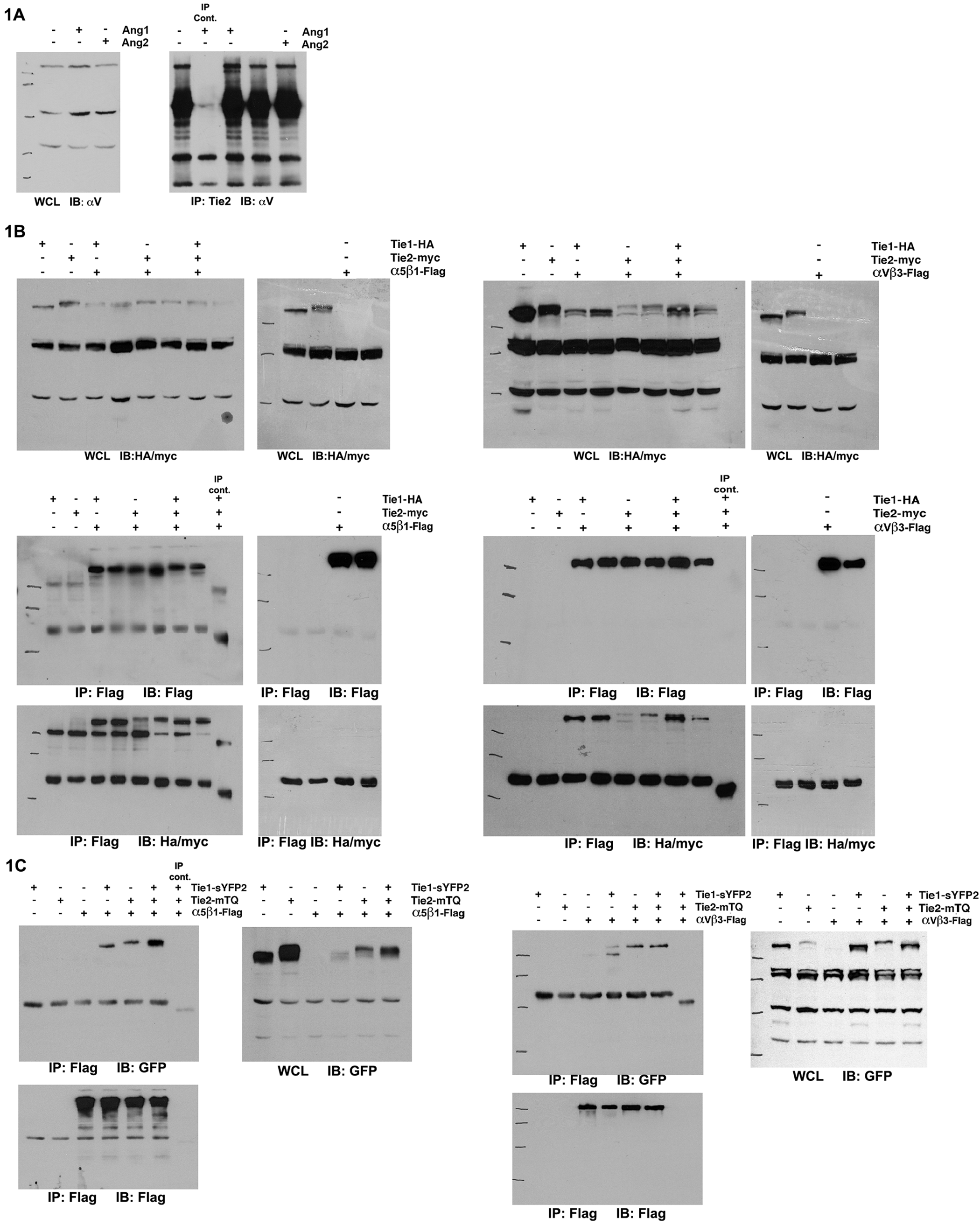

Supplement: S6 Fig — (TIF) [file pone.0163732.s006.tif]

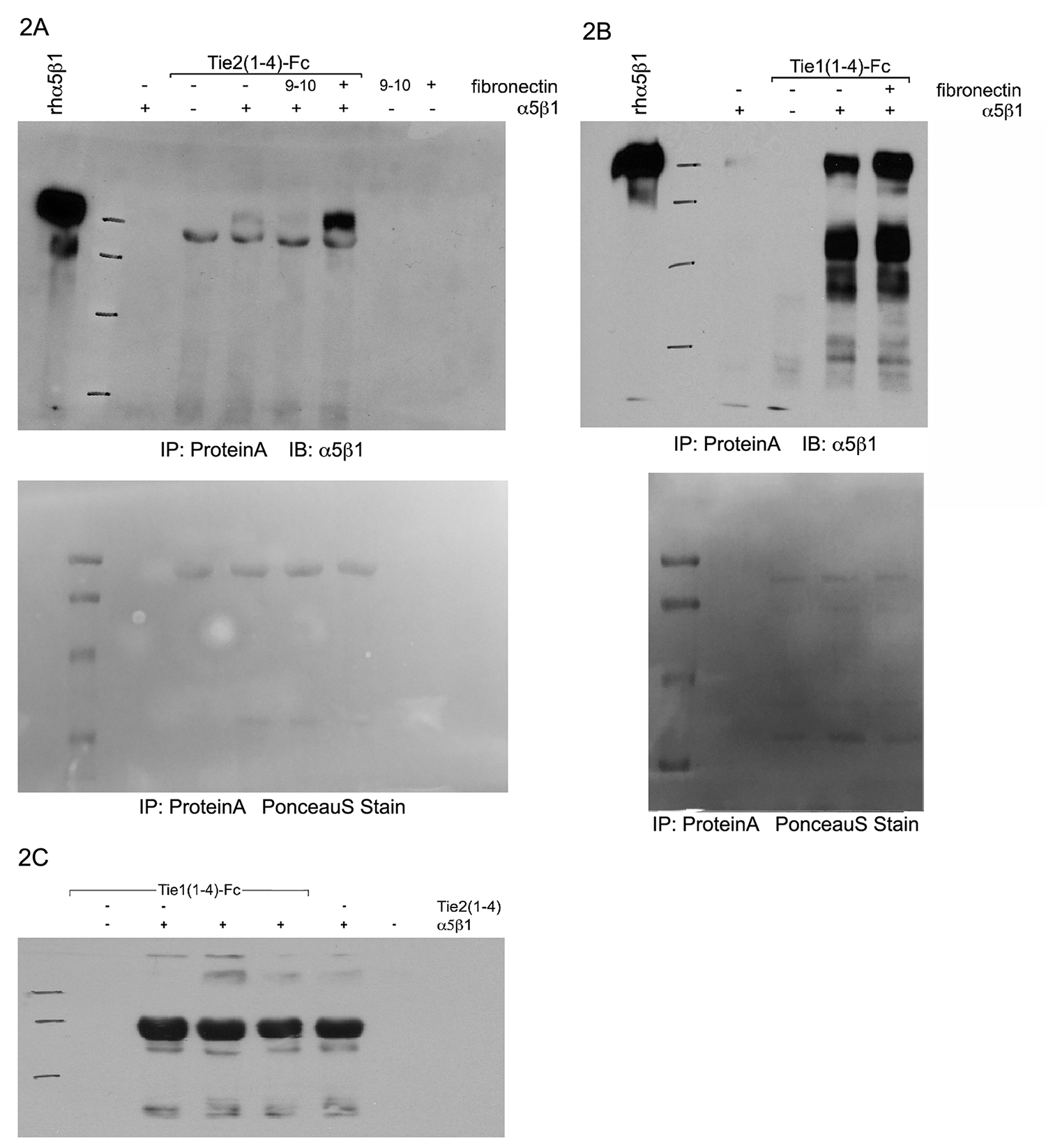

Supplement: S7 Fig — (TIF) [file pone.0163732.s007.tif]

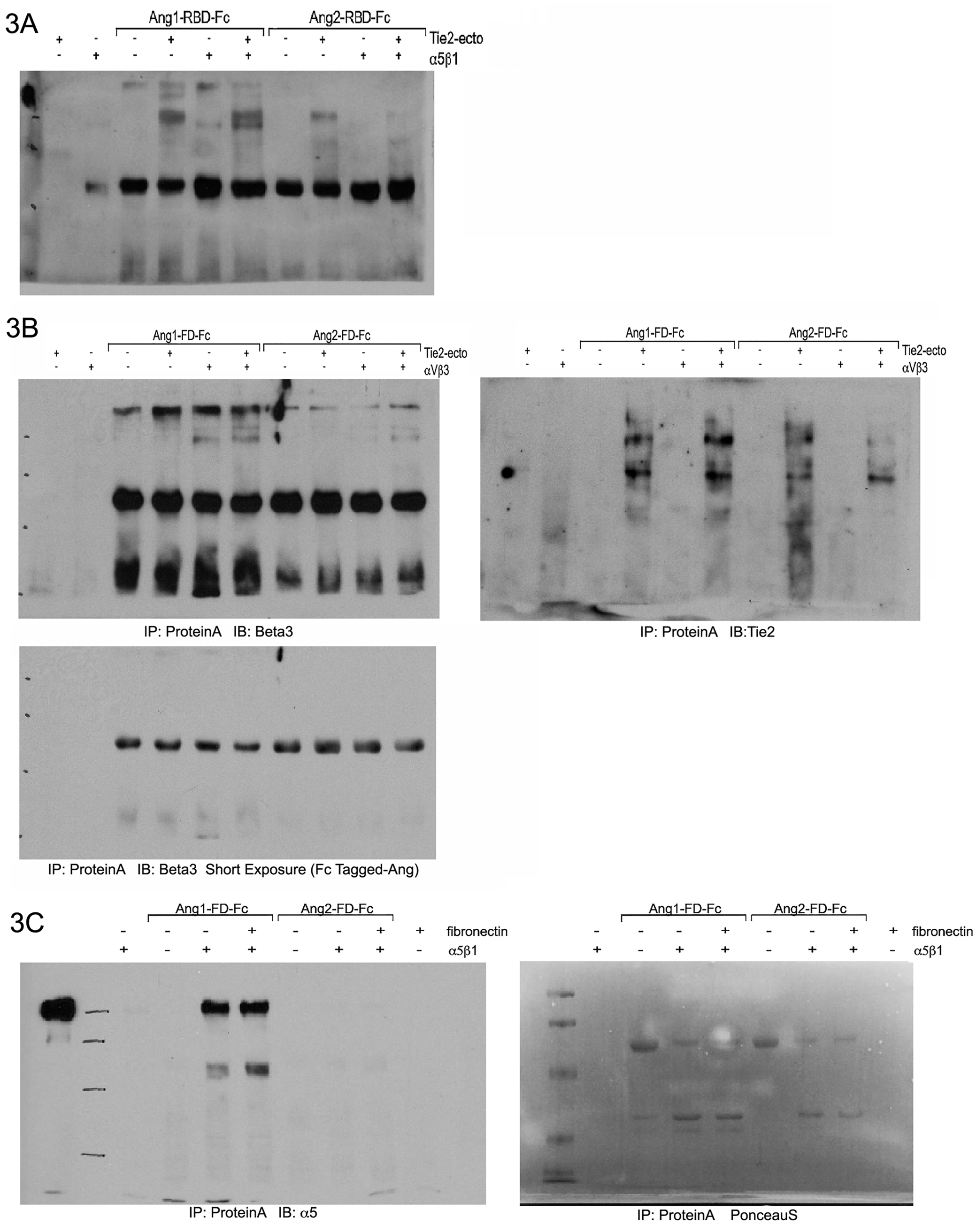

Supplement: S8 Fig — (TIF) [file pone.0163732.s008.tif]

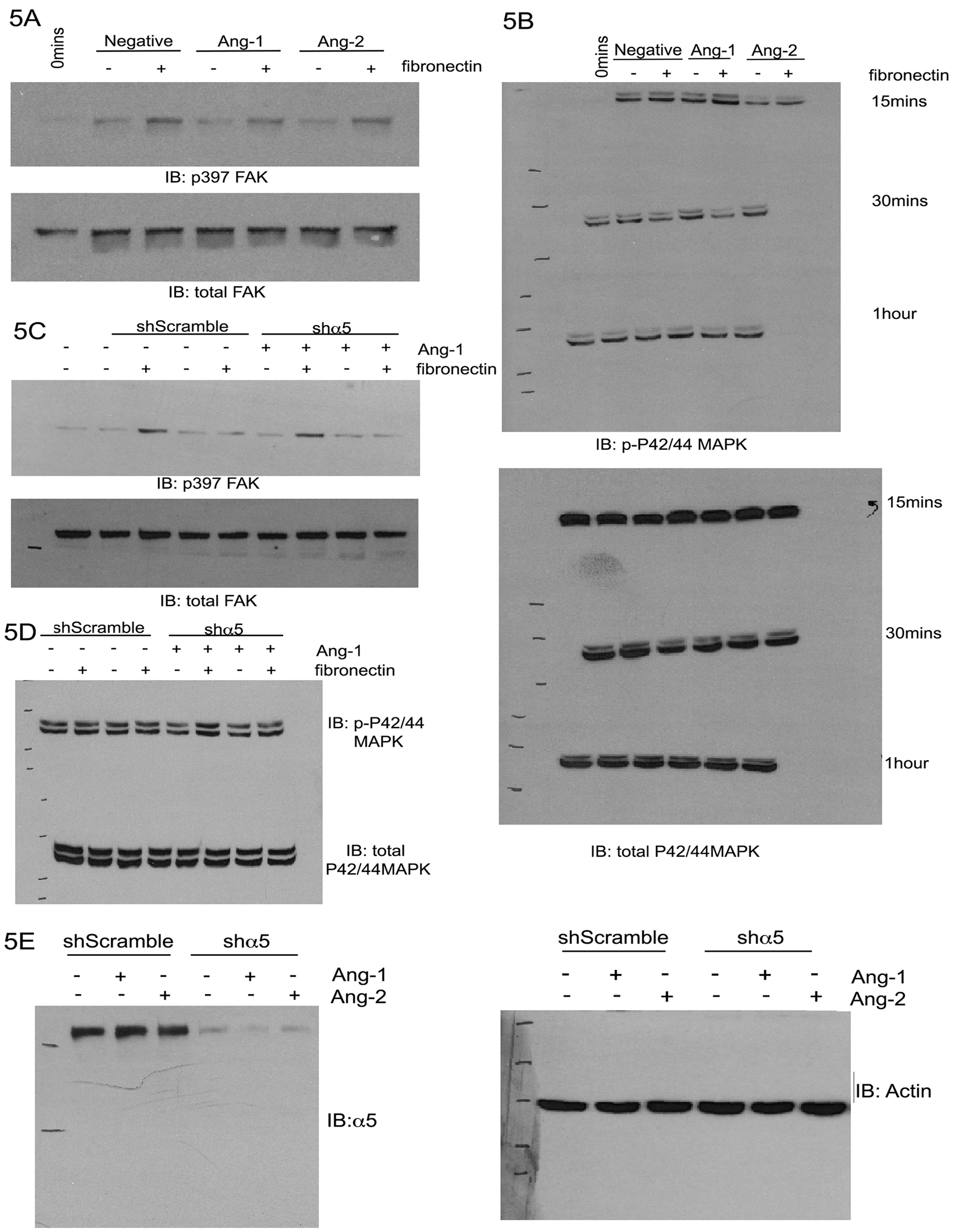

Supplement: S9 Fig — (TIF) [file pone.0163732.s009.tif]

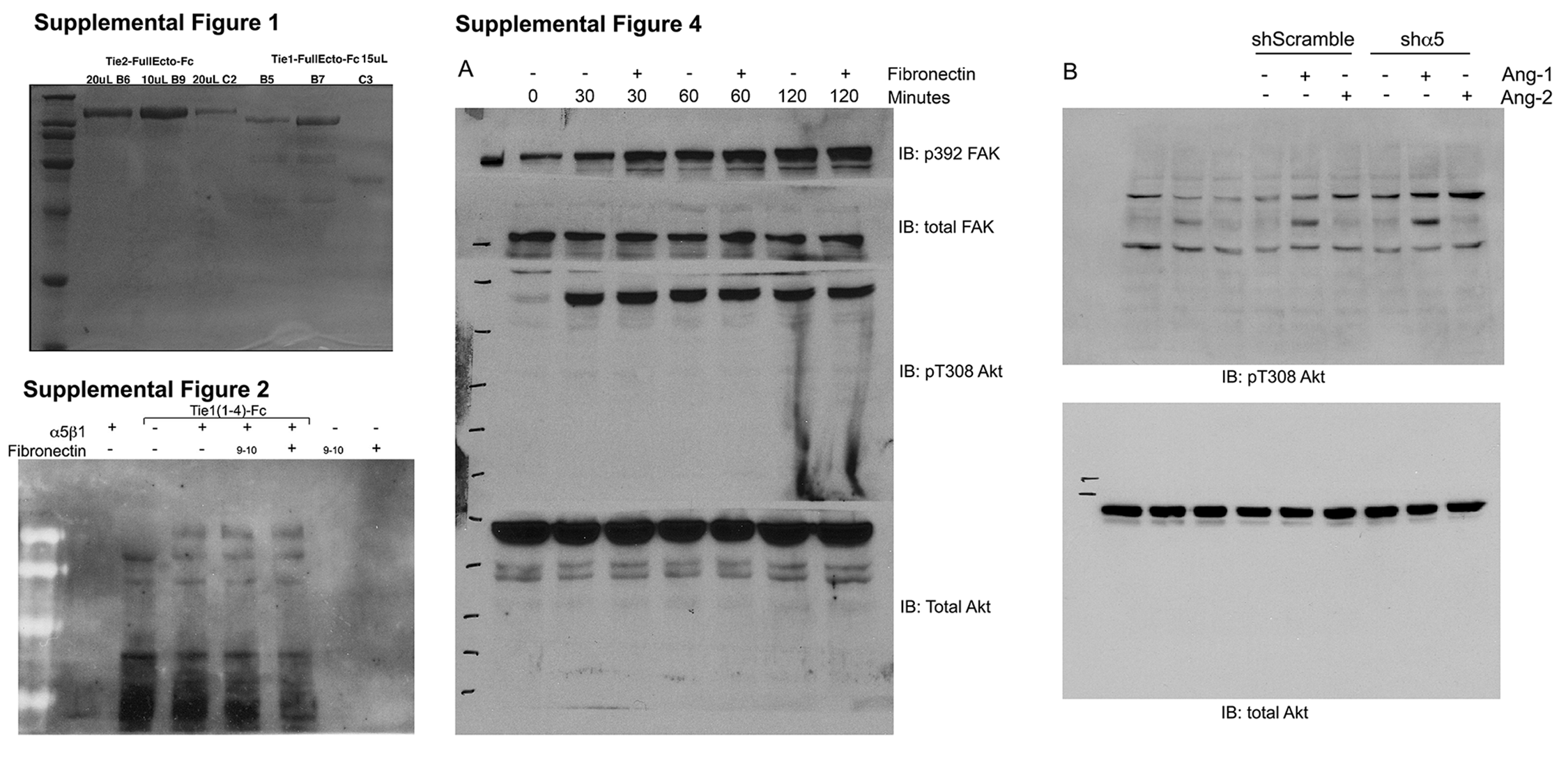

Supplement: S10 Fig — (TIF) [file pone.0163732.s010.tif]
