# Supplementary figures and images for: Correction: Constitutive Association of Tie1 and Tie2 with Endothelial Integrins is Functionally Modulated by Angiopoietin-1 and Fibronectin
Source: PLoS One. 2017 May 31;12(5):e0179059. doi: 10.1371/journal.pone.0179059 (PMC5451130; doi:10.1371/journal.pone.0179059)

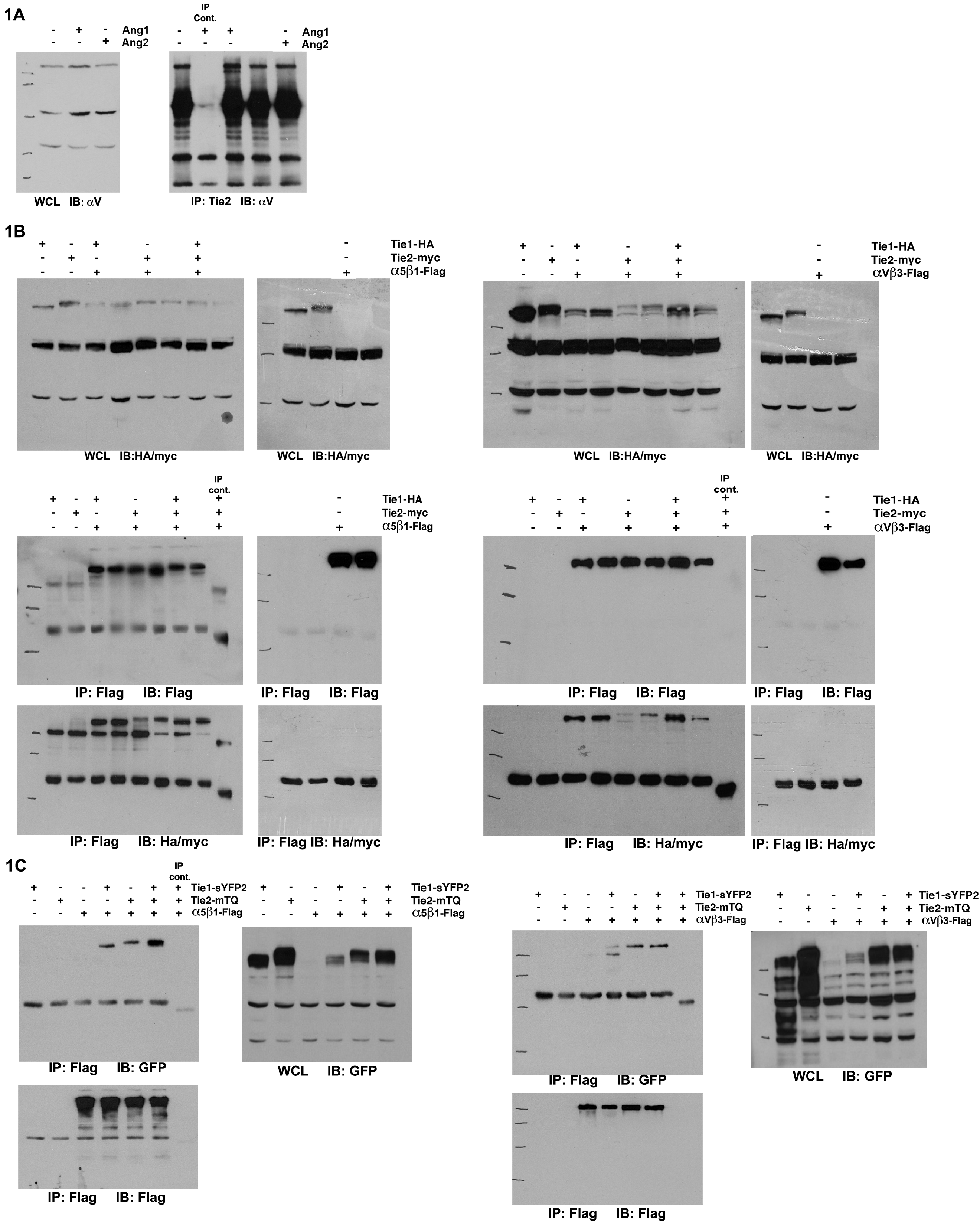

Supplement: S6 Fig — (TIF) [file pone.0179059.s001.tif]
